# *Mycobacterium tuberculosis* Methyltransferase Rv1515c Can Suppress Host Defense Mechanisms by Modulating Immune Functions Utilizing a Multipronged Mechanism

**DOI:** 10.3389/fmolb.2022.906387

**Published:** 2022-06-24

**Authors:** Anshu Rani, Anwar Alam, Faraz Ahmad, Manjunath P., Abhinav Saurabh, Sheeba Zarin, Dipendra Kumar Mitra, Seyed E. Hasnain, Nasreen Z. Ehtesham

**Affiliations:** ^1^ Kusuma School of Biological Sciences, Indian Institute of Technology Delhi (IIT-D), New Delhi, India; ^2^ ICMR-National Institute of Pathology, Safdarjung Hospital Campus, New Delhi, India; ^3^ Department of Transplant Immunology and Immunogenetics, All India Institute of Medical Sciences, New Delhi, India; ^4^ Department of Biochemical Engineering and Biotechnology, Indian Institute of Technology Delhi (IIT-D), New Delhi, India; ^5^ Department of Life Science, School of Basic Sciences and Research, Sharda University, Greater Noida, India

**Keywords:** macrophage, multi drug resistance, tuberculosis, tregs, pathogenicity

## Abstract

*Mycobacterium tuberculosis* (*M. tb*) gene *Rv1515c* encodes a conserved hypothetical protein exclusively present within organisms of MTB complex and absent in non-pathogenic mycobacteria. *In silico* analysis revealed that Rv1515c contain S-adenosylmethionine binding site and methyltransferase domain. The DNA binding and DNA methyltransferase activity of Rv1515c was confirmed *in vitro*. Knock-in of Rv1515c in a model mycobacteria *M. smegmatis* (*M. s*_Rv1515c) resulted in remarkable physiological and morphological changes and conferred the recombinant strain with an ability to adapt to various stress conditions, including resistance to TB drugs. *M. s*_Rv1515c was phagocytosed at a greater rate and displayed extended intra-macrophage survival *in vitro*. Recombinant *M. s*_Rv1515c contributed to enhanced virulence by suppressing the host defense mechanisms including RNS and ROS production, and apoptotic clearance. *M. s*_Rv1515c, while suppressing the phagolysosomal maturation, modulated pro-inflammatory cytokine production and also inhibited antigen presentation by downregulating the expression of MHC-I/MHC-II and co-stimulatory signals CD80 and CD86. Mice infected with *M. s*_Rv1515c produced more Treg cells than vector control (*M. s*_Vc) and exhibited reduced effector T cell responses, along-with reduced expression of macrophage activation markers in the chronic phase of infection. *M. s*_Rv1515c was able to survive in the major organs of mice up to 7 weeks post-infection. These results indicate a crucial role of Rv1515c in *M. tb* pathogenesis.

## Introduction

Tuberculosis (TB), a life-threatening disease caused by *Mycobacterium tuberculosis (M. tb),* infects nearly a third of the world population (Chakaya et al., 2021; WHO, 2021). Around one-fourth of the world population is infected with latent TB (Houben and Dodd, 2016). There is no vaccine available today for TB except BCG, developed by Calmette and Guerin in 1921, which itself is ineffective in preventing pulmonary TB in adults. Rapid emergence of multidrug resistance strains has highlighted the need to explore virulence factors in *M. tb* that have allowed this bacteria to evolve as one of the most successful pathogen known to mankind.

The major focus in determining the virulence factors has been on the Regions of Differences (RD) in the *M. tb* genome which are deleted in *M. bovis* BCG. 16 RD locus are reported to be present in *M. tb* (Yang et al., 2021) and these are known to harbour essential genes for virulence and pathogenicity. While RD6 was found to be highly variable among the clinical isolates, *M. tb/M. bovis* specific RD1, RD2, and RD3 regions were reported to be deleted in BCG (Mahairas et al., 1996; Rao et al., 2005). Exploring the function of the RD6 region of *M. tb* may help in understanding its role in *M. tb* pathogenesis and to search for novel therapeutic avenues in TB.

*M. tb* has evolved mechanisms that allow it to infect and survive within the host environment and evade the immune system. This requires interplay of several virulence factors that allow the *M. tb* to adapt to the host immune challenges. Horizontal transfer of new genes increased the pathogenic potential of slow-growing *M. tb* and permitted its adaptation to the lungs (Rosas-Magallanes et al., 2006; Rosas-Magallanes et al., 2007; Jang et al., 2008). We earlier reported the presence of 121 methyltransferases (Mtases) in *M. tb* H_37_Rv, which was much higher compared to other pathogenic, non-pathogenic and opportunistic species of mycobacteria (Grover et al., 2016). We identified two *M. tb* MTases, Rv1509, and Rv1515c, present exclusively in the MTB complex organisms i.e., *M. africanum and M. canettii* (Grover et al., 2016)*.* In a comprehensive *M. tb* and BCG membrane proteome analysis it was shown that the Rv1515c protein, associated with RD4 region, is present only in the membrane fraction of *M. tb* (Gunawardena et al., 2013).

In the present study, we examined the functional role of hypothetical protein Rv1515c of *M. tb*. The effect of Rv1515c on cellular properties including morphology, resistance to stress, virulence and immune response were investigated. The role of Rv1515c in immune modulation and pathogenesis was assessed *in vivo* by scoring gain of functions following insertion into the non-pathogenic bacteria. Our study provides crucial insights on the role of Rv1515c in host-pathogen interaction during TB pathogenesis.

## Materials and Methods

### Cloning, Expression, and Purification of Recombinant Rv1515c

The ORF encoding *Rv1515c* gene was PCR amplified from H_37_Rv genomic DNA (BEI Resources, United States) using forward primer CTT​GGA​TCC​ATG​TCG​ACA​AAC​CCA​GGA and reverse primer CCT​AAG​CTT​TCA​CCG​GGT​CTT​GAT​ACC. The gene *Rv1515c* was cloned in bacterial expression vector pET28a^+^ using restriction sites *Bam*HI and *Hin*dIII. Positive clones were screened by colony PCR, confirmed through restriction digestion and the recombinant clone was transformed into both *E. coli* BL21 (DE3) and Clearcoli BL21 (DE3) strain. 1 mM IPTG (Iso-propyl thiogalactosidase) was added to the culture to induce overexpression of Rv1515c. The recombinant protein Rv1515c was purified using Ni-NTA affinity chromatography, eluted in buffer composed of 50 mM Tris buffer, 300 mM imidazole and properly dialysed in 50 mM Tris buffer for further experiments. SDS-PAGE of protein was done and confirmed by western blot using anti-His tagged mouse antibody.

### DNA Binding and Methyltransferase Activity of Rv1515c

The DNA binding activity of purified recombinant Rv1515c protein at different concentrations of DNA (H_37_Rv, BEI resources) was assessed using fluorescence spectroscopy. Rv1515c protein in 20 mM Tris buffer (pH = 8.5) solution was excited at 280 nm and the emission spectrum was assessed from 300 to 450 nm. Fluorescence titration experiment was performed at a constant Rv1515c protein concentration in presence of increasing concentration of plasmid DNA concentration (20–200 ng) and fluorescence spectra were plotted.

The SAM-dependent DNA methyltransferase (Mtase) activity of Rv1515c was assessed using the EpiQuik DNA MTase activity assay kit (EPIGENTEK, United States), as per the manufacturer’s protocol. In brief, purified recombinant Rv1515c protein was incubated for 1.5 h with cytosine rich DNA substrate. Bovine serum albumin (BSA) was used as a negative control. 5-methylcytosine capture antibody was added after washing the wells and incubated for 1 h at room temperature. The plate was washed, detection antibody was added and incubated for 30 min. After adding colour developing solution it was incubated in dark at room temperature. After the development of colour, the enzyme reaction was stopped and absorbance was measured spectrophotometrically at 450 nm.

### Construction of Recombinant Strains

The *Rv1515c* gene, obtained from pET28a^+^ *Rv1515c* construct using restriction sites *Bam*HI and *Hin*dIII, was sub-cloned in Mycobacterium constitutive expression vector pST-Ki. *Mycobacterium smegmatis* mc^2^155 cells, grown in 7H9 media supplemented with 10% Oleic- Albumin-Dextrose-Catalase (OADC) and 0.05% Tween 80, were harvested at OD_600_ of 0.4–0.6 and used for competent cell preparation. *M. smegmatis* mc^2^155 was electroporated with pST-Ki-Rv1515c or pST-Ki to generate recombinant (*M. s*_Rv1515c) or vector control (*M. s*_Vc) strains, respectively. Recombinant and vector control cells were incubated in 7H9 broth at 37°C for 3 h and plated on 7H11 agar supplemented with OADC containing 15 µgml^−1^ kanamycin (Sigma Aldrich). Colony PCR was used for screening the transformed colonies of *M. smegmatis*.

### Expression Confirmation of Rv1515c Gene in *M. smegmatis*

*M. s*_Rv1515c and *M. s*_Vc were cultured in 7H9 complete broth supplemented with 50 μg/ml kanamycin for 24 h. Cell pellet was obtained by centrifugation (5000 rpm, 10 min) and washed with PBS. The cell pellet was dissolved in SDS-PAGE loading dye and heated at 95°C for 30 min. The lysed fractions were fractionated by electrophoresis in 10% SDS-PAGE and Rv1515c protein expression was confirmed by western blot.

### Colony Morphology

*M. s*_Vc and *M. s*_Rv1515c cells in the log phase of growth were examined microscopically after acid-fast staining for purity and clumping of the bacteria. *M. s*_Vc and *M. s*_Rv1515c cells were also plated on a 7H11 agar plate and incubated at 37°C for 3 days to assess colony morphology. For scanning electron microscopy (SEM) and transmission electron microscopy (TEM), cells were processed as per standard protocols (Wisse et al., 2010). SEM imaging (at 20kX magnification) was done using XEISS scanning electron microscope and the length of the bacteria was analysed using XEISS software. TEM analysis was done using the Hitachi transmission electron microscope. For atomic force microscopy (AFM), desiccated samples were imaged at 13 kHz, force constant 0.2 N/m and data were analysed using Gwyddion software. All the imaging data were plotted using GraphPad Prism 5.

### Analysis of Cellular Aggregation and Cell Wall Permeability

Bacterial cultures in the log phase of growth were kept in static condition for 1 h to allow the cell aggregates to settle down at the bottom surface. The cell aggregates were subjected to Hexadecane partitioning assay, as per the standard protocols (Jankute et al., 2017), to assess hydrophobicity of the bacterial cells. Hydrophobicity index was calculated as the percentage of bacterial suspension (OD_600_) in the PUM buffer after separating the two phases.

To assess cell permeability, *M. s*_Vc and *M. s*_Rv1515c cultures were washed with PBS, stained with EtBr (2 μg/ml) or Nile Red (20 μM) for 1.5 h and the unbound dyes were washed off. The amounts of EtBr and Nile Red that were taken by the cells were measured using fluorescence spectrometry at 544 and 590 nm, respectively (Abdalla et al., 2020).

### Survival Assay of Bacteria Cultured Under Different Stress Conditions

The recombinant and vector control bacterial cells were cultured until the log phase and the cultures of OD_600_ were equalized to an equal number of CFU. Cells were treated with SDS (0.1%), lysozyme (500 ug/ml) and mild acid (pH 5) stress. The cultures were incubated at 37°C for up to 48 h, plated at different serial dilutions onto LB agar plates and incubated at 37°C for 3 days to determine the CFU counts. For assessing the minimum inhibitory concentration (MIC) against first line anti-TB drugs, *M. s*_Vc and *M. s*_Rv1515c cells (OD_600_∼0.4), were diluted up to ∼1,000 fold and inoculated in 96-well plates. Anti-TB drugs (ethambutol, Isoniazid, and streptomycin) were serially diluted in 7H9 broth and added to the culture wells. The plates were incubated in a shaking incubator (100 rpm/min) for 40 h. After incubation, resazurin solution (0.3%) was added to each well and the plate was incubated for another 8 h. The reduction of oxidized blue resazurin to a red dye resorufin due to activity of the live cells was assessed spectrophotometrically at 570 nm. The MIC of *M. s*_Rv1515c and *M. s*_Vc cultures treated with anti-TB drugs were compared with the corresponding *M. s*_ Rv1515c and *M. s*_Vc cultures, untreated with anti-TB drugs.

### Assay for Intracellular Survival and Localization of Bacteria

RAW 264.7 murine macrophages (0.5 × 10^6^ cells) were cultured in 24-well plate in the presence of DMEM media (Sigma Aldrich) supplemented with 10% (v/v) fetal bovine serum (FBS) and streptomycin/Penicillin sodium G mixture (both from Sigma Aldrich), hereafter called the complete media. Control *M. s*_Vc and recombinant *M. s*_Rv1515c cells were washed with PBS and resuspended in DMEM. The resuspended cultures of control and recombinant *M. smegmatis* were passed 4–5 times through a syringe needle (23 gauge) to obtain single-cell suspension. RAW 264.7 cells were incubated with equal number of *M. s*_Vc and *M. s*_Rv1515c cells at a multiplicity of infection (MOI) of 1:10 for 4 h. Thereafter, the RAW 264.7 macrophages were washed three times with PBS to remove extracellular bacteria and complete DMEM media containing gentamycin (2% v/v) was added to kill the extracellular bacteria. Cells were lysed and the bacteria that were internalized after 4 h were plated at different serial dilutions on LB agar plate and incubated at 37°C for 3 days to determine the CFU count. Macrophages were also replated in culture discs and post infection the infected macrophages were lysed with ice-cold PBS at 24, 48, and 72 h post-infection. The intracellular bacteria that survived were estimated by CFU count, as mentioned above. Bacteria stained with dyes were used to assess uptake and survival in the macrophage, which were also assessed microscopically and by flow cytometry.

### RNA Isolation, cDNA Synthesis, and Real-Time PCR

RAW 264.7 macrophages were infected with *M. s*_Vc or *M. s*_Rv1515c at a MOI 1:10. After 4 h of infection, macrophages were washed with PBS and complete DMEM with gentamycin was added to the wells. After 24 and 48 h of post infection, RAW 264.7 cells were collected for RNA isolation. RNA was extracted using the TRIzol reagent (Invitrogen) followed by isopropanol precipitation. The cDNA was synthesized from 1 μg RNA using iScript cDNA Synthesis Kit (Biorad, Cat. No. 1708891). The resulting cDNA was used to amplify mRNA levels of TNFR1, TNFR2, Cathelicidin (CRAMP), TACE, and VDAC1 using iTaq universal SYBR Green Supermix (Biorad, Cat No. 1725120) by real-time quantitative Polymerase Chain Reaction (RT-PCR) and normalised with the β-actin expression level. The list of primers used for RT-PCR reactions is provided in Supplementary Table S1.

### Cytokine ELISA and Determination of NO/ROS

RAW 264.7 cells (0.5 × 10^6^ cells) were incubated with equal number of *M. s*_Vc and *M. s*_Rv1515c cells at a MOI of 1:10. After 24 and 48 h post-infection, the culture supernatant was collected and ELISA was done to assess the levels of TNF-α, IL-6, and IL-12, as per the manufacturer protocol (Biolegend, United States). 50 μl of Griess reagent (Thermofisher Scientific, United States) was added to 150 μl of culture supernatants that were collected at 24 or 48 h and incubated for 30 min in dark. Level of NO was measured spectrophotometrically at 545 nm. For ROS measurement, cells were stained with CellROX green (Thermofisher Scientific, USA) at 5 μM concentration and incubated at 37°C for 30 min in the dark. Cells were then washed with PBS and analysed flow cytometrically.

### *In Vitro* Infection Study for Assessing Apoptosis, Phagolysosomal Markers, and Intracellular Localization of Bacteria

RAW 264.7 cells, infected with *M. s*_Vc and *M. s*_Rv1515c at MOI of 1:10, were incubated for 4 h. Post infection, RAW 264.7 cells were washed with PBS and treated with complete DMEM media containing gentamycin to kill the extracellular bacteria. Infected RAW 264.7 cells (2 × 10^6^ cells) were again re-seeded/incubated in complete DMEM media for 24, 48 h. Staurosporine treated RAW264.7 cells were used as positive control. At the end of incubation period, RAW 264.7 cells were detached using ice-cold PBS, washed with PBS buffer and stained with FITC-Annexin V Apoptosis Detection Kit (BD Biosciences, United States). Apoptotic cells were acquired using BD FACS (LSR Fortessa) instrument and analysed using FlowJo software.

Western blot of infected RAW 264.7 cells lysates, obtained at 24 and 48 h post-reseeding, were used for western blot analysis to assess the expression of Rab5, Rab7, and B-actin (as control). All the antibodies were procured from Cell Signalling Technologies. PVDF membranes were developed using Takara chemiluminescence reagent.

*M. s*_Rv1515c and *M. s*_Vc were labelled with FITC dye and used for intracellular localization experiments. RAW 264.7 macrophages, infected with bacteria at MOI of 1:10, were seeded on coverslips in 24-well plate. After 4 h of infection, the extracellular bacteria was washed as described above. Lysotracker (Invitrogen) was used to stain the lysosomes in RAW 264.7 cells and mounted with antifade reagent. Cells (40X magnification) were visualized using fluorescent microscope (ZEISS).

### Mice Study

The study was carried out as per the guidelines of CPCSEA, India and approved by ICMR-NIP Institutional Animal Ethics Committee. C57BL/6 female mice (6–8 week) were procured from All India Institute of Medical Sciences (AIIMS) central animal facility, New Delhi. Animals were acclimatized for 1 week prior to experiments. Animals were provided free access to complete pelleted food, water, and 12-h photoperiod.

C57BL/6 mice (*n* = 6) were intraperitoneally administered with *M. s*_Vc and *M. s*_Rv1515c bacteria at 5 × 10^7^ CFU. Mice were sacrificed at different time points- 1, 3, and 7 weeks and the organs (spleen, pancreas, liver, and lungs) were isolated. The organs were homogenized in PBS and serially diluted on 7H11 agar plates for CFU enumeration.

### Flow Cytometry

For *in vitro* studies, RAW 264.7 cells were infected with *M. s*_Vc and *M. s*_Rv1515c, as described above. The expression of antigen presentation molecules (MHC I, MHC II) and co-stimulatory molecules (CD80 and CD86) were studied. For *in vivo* studies, the peritoneal macrophages and lymph nodes were isolated from mice infected with *M. s*_Vc and *M. s*_Rv1515c. Uninfected mice were used as controls. Antibodies and reagents used for flow cytometry were obtained from Biolegend, United States. For extracesllular staining of RAW 264.7 cells or lymphocyte subpopulations, single cell suspension (0.2 × 10^6^ cells) of peritoneal macrophages and lymph nodes were stained with F_c_ block and incubated for 10–15 min at 4°C. The surface marker cocktail was added and incubated at 4°C for 30 min. Subsequently, cells were washed twice with 0.5 ml staining buffer, fixed with fixation solution and analysed through flow cytometer (LSR Fortessa, BD Biosciences).

### Histopathology

Immediately after the dissection of mice, organs were stored in 10% neutral buffered formalin for fixation, as per standard protocols. Briefly, samples were processed using an automated tissue processor (Microm International GmbH, Germany) and serially dehydrated (TEC2800 cryoconsole). The paraffinized tissue was excised into a 5 μm thickness section using a microtome (Leica Biosystems). Tissue sections were placed on glass slides and kept for 10 min on a heating block for deparaffinisation. Subsequently, tissue sections were dipped in Xylene, then in acetone and then rehydrated using 100% ethanol. For staining, sample was treated with haematoxylin for 20 min, decolourised with acid alcohol, washed with running water, dipped in ammonium water and washed. For eosin staining, sample was incubated for 2–3 min with eosin and dipped in absolute ethanol followed by acetone wash for 5 min and then dipped in xylene. Stained samples were finally mounted with DPX.

### Statistical Analysis

All experiments were performed in triplicate. All the data were plotted using GraphPad Prism 5 software, and statistical significance was determined using ANOVA and t-Test. *p* < 0.05 was considered significant. The different levels of significance considered were as follows: *p < 0.05, **p < 0.01, ***p < 0.001, and ****p < 0.0001.

## Results

### Rv1515c Displayed DNA Binding and DNA Methyltransferase Activity

**FIGURE 1:**
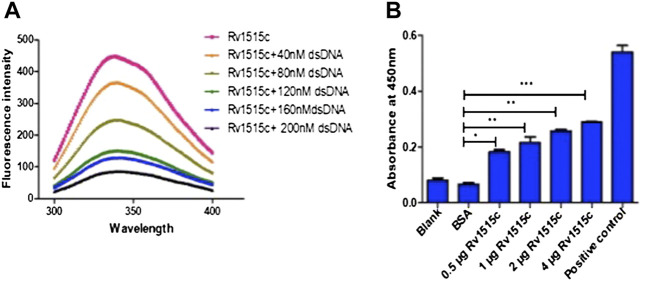
Biochemical characterization of *M. tb* Rv1515c. (**A)** Purified recombinant Rv1515c protein was titrated with 40, 80, 120, 160, and 200 nM of DNA and the fluorescence spectra was plotted. **(B)** 0.5, 1, 2, and 4 μg of purified recombinant Rv1515c protein was incubated with DNA. BSA was used as negative control and kit based reagent was used as positive control, respectively. DNA methyltransferase activity was measured as described in methods. Data are represented as mean ± SD from three independent experiments and analysed by one-way ANOVA, followed by Bonferroni test. *p < 0.05, **p < 0.01, ***p < 0.001.

*In silico* pBLAST analysis and I-Tasser based prediction of the structure of *M. tb* hypothetical protein Rv1515c, showed presence of S-adenosyl methionine (SAM) binding site at the N-terminal of protein and MTase domain, as reported previously (Yang et al., 2021). The SAM-dependent MTase was conserved from 68–163 amino acid in the protein sequence (Supplementary Figure S1). In order to assess the physical interaction of Rv1515c protein with DNA, Rv1515c was titrated with different concentrations of DNA and the fluorescence spectra was examined, as described in methods. Upon binding to DNA, the intrinsic fluorescence intensity quenched with increasing concentration of DNA (Figure 1A), thereby confirming the interaction of Rv1515c protein with DNA. The methyltransferase activity of Rv1515c was biochemically characterized using a methylated cytosine targeted DNA MTase kit. DNA MTase enzyme transfers a methyl group from Adomet to cytosine and methylates the DNA. The methylated DNA was detected using a 5-methylcytosine antibody and enzyme activity was quantified. Rv1515c displayed concentration-dependent increase in DNA methyltransferase activity (Figure 1B). These results suggest that *M. tb* Rv1515c, like other DNA methyltransferases, exhibits robust DNA binding activity *in vitro* and methylates DNA at the cytosine position.

### Knock-In of Rv_1515c in *M. smegmatis* Leads to Change in Cell Size, Cell Wall Morphology, and Increased Cellular Aggregation

**FIGURE 2:**
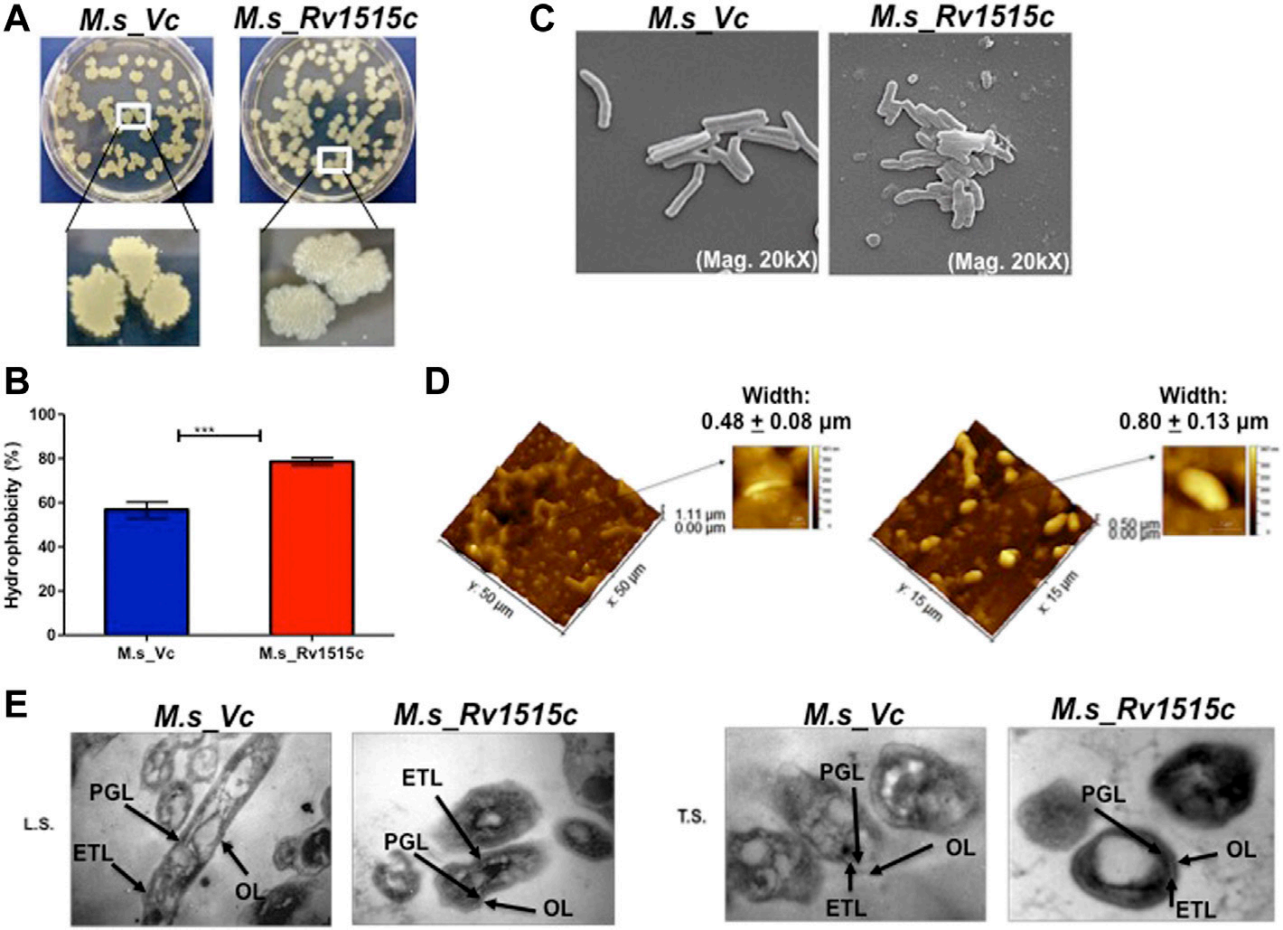
Knock-in of *Rv1515c* gene in *M. smegmatis* modulates colony morphology, cell surface hydrophobicity and cell dimensions. **(A)** *M. s*_Vc and *M. s*_Rv1515c cells were plated on a 7H11 agar plate and incubated at 37°C for 3 days to allow colony formation. **(B)** *M. s*_Vc and *M. s*_Rv1515c cells pellets were subjected to Hexadecane partitioning assay and cell surface hydrophobicity of bacterial cells were analyzed. **(C)** SEM imaging (20kX) of *M. s*_Vc and *M. s*_Rv1515c for assessing the length of bacteria. SEM data were analyzed with XEISS software. **(D)** AFM imaging to analyze the topology of *M. s*_Rv1515c and *M. s*_Vc cells. Inset is the enlarged three-dimensional view of bacterial surface topology. AFM data were analyzed by Gwyddion software. **(E)** TEM anslysis of Ultrathin structure (LS: longitudinal section; TS: transverse section) of *M. s*_Vc and *M. s*_Rv1515c revealed cell wall modification. OL (Outer layer), PG (Peptidoglycan), and ETL (Electron transparent layer). Data are represented as mean ± SD of from three independent experiments, and statistical analysis was performed using a two-tailed unpaired t-test. ***p < 0.001.

In order to understand the role of Rv1515c on cellular phenotype, Rv1515c was cloned and expressed in a non-pathogenic strain *M. smegmatis.* Recombinant *M. smegmatis* (*M. s*_Rv1515c) constitutively expressing Rv1515c showed a significant decrease in growth as compared to the wild type (*M. s*_WT) or vector control (*M. s*_Vc) (Supplementary Figure S2). Equal number of *M. s*_Rv1515c and *M. s*_Vc cells were taken for all experiments by carefully monitoring the culture OD and CFU. The colony size and morphology of *M. s*_Rv1515c were compared with *M. s*_Vc by culturing the bacteria on LB agar plates. We observed that *M. s*_Rv1515c colonies were smaller in size and showed rough surface as compared to the *M. s*_Vc colonies that were large and possessed smooth surface (Figure 2A). Smaller colonies in *M. s*_Rv1515c were indicative of slow growth, which was in line with our observed growth kinetic for the Rv1515c knock-in *M. smegmatis*. After incubation for 3 days on the LB agar plate, we observed moist, granular, and waxy colony morphology of *M. s*_Rv1515c. These observations suggest that knock-in of *M. tb Rv1515c* gene affected the phenotype of the recombinant *M. smegmatis*.

*M. s*_Rv1515c and *M. s*_Vc were cultured in LB broth and their aggregation in aqueous media were studied to assess the change in surface hydrophobicity of the recombinant strains. It was observed that *M. s*_Rv1515c aggregated in static aqueous media as compared to the *M. s*_Vc (Supplementary Figure S2B), suggesting that knock-in of *M. tb Rv1515c* gene in *M. smegmatis* resulted in change in the hydrophobicity of cell surface in the recombinant strain of *M. smegmatis* as compared to the vector control cells. A hexadecane partitioning assay showed that the hydrophobicity of cell surface was significantly (*p* < 0.001) increased in *M. s*_Rv1515c as compared to the *M. s*_Vc (Figure 2B), which may be due to changes in lipid composition of the cell wall and increased production of hydrophobic lipids. Rv1515c, therefore may play an important role in increasing the hydrophobicity of mycobacterial cell wall.

Microscopic examination of acid fast stained bacteria showed that *M. s*_Rv1515c was smaller in size and exhibited clumping as compared to the *M. s*_Vc (data not shown). In-depth analysis of the changes in the mycobacterial cell morphology was done using Electron microscopy. SEM revealed that the mean cell length of control *M. s*_Vc was 2.94 ± 0.78 μm while the mean cell length of *M. s*_Rv1515c was 1.42 ± 0.33 μm (Figure 2C). The changes in the surface topological structure due to insertion of *Rv1515c* gene in *M. smegmatis* were determined at the nanoscale level using AFM. We observed irregular surface topology in *M. s*_Rv1515c as compared to the *M. s*_Vc*.* The mean bacterial width also significantly (*p* < 0.001) increased from 0.48 ± 0.08 μm in *M. s*_Vc to 0.80 ± 0.13 μm in *M. s*_Rv1515c (Figure 2D). TEM analysis also showed that *M. s*_Rv1515c was broader in diameter and had bulged-out structure on the cell wall as compared to the *M. s*_Vc, which might be due to the deposition of extra lipids in the cell wall. *M. s*_Rv1515c exhibited increased electron-dense layer as compared to *M. s*_Vc (Figure 2E), indicating change in cell wall composition. These results suggests that Rv1515c is crucial for remodelling of the cell wall of *M. tb* and may affect the ability of the bacterium to resist stress.

### Rv1515c Modulates Cell Wall Permeability Which Confers Resistance to Stress

**FIGURE 3:**
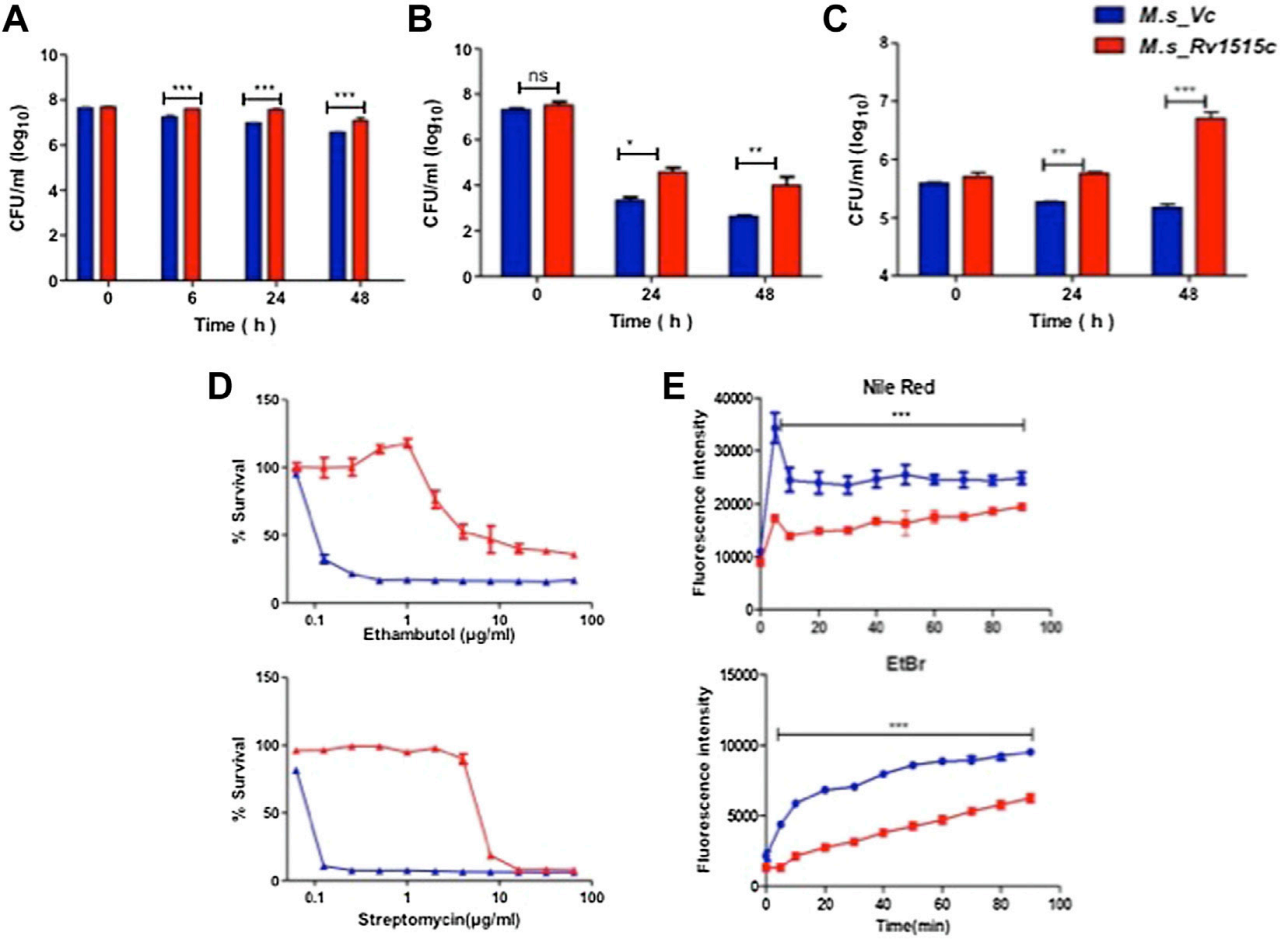
Recombinant *M. smegmatis* expressing *M. tb* Rv1515c displayed greater resistance to various stress conditions and altered cell wall permeability. Growth of *M. s*_Vc and *M. s*_Rv1515c in presence of **(A)** 0.1% SDS; **(B)** 500 μg/ml Lysozyme; **(C)** at pH 5; and **(D)** serial dilutions of anti-TB drugs Ethambutol and Streptomycin were estimated. **(E)** Uptake of Nile red (20 μM) and Ethidium Bromide (2 μg/ml) by *M. s*_Vc and *M. s*_Rv1515c cells for evaluating cell permeability. Data are represented as mean ± SD from three independent experiments. Statistical analysis was performed using two-way ANOVA followed by Bonferroni test. **p < 0.01, ***p < 0.001.

In order to understand the effect of altered cell wall morphology in conferring resistance to stress, *M. s*_Vc and *M. s*_Rv1515c were cultured in presence of SDS (0.1%), mild acid (pH 5), lysozyme, and anti-TB drugs (ethambutol, Isoniazid, and streptomycin). In presence of SDS, a detergent that denatures proteins, *M. s*_Rv1515c exhibited up to 9.4, 11, and 9.2 fold more survival as compared to the *M. s*_Vc at 6, 24, and 48 h post-SDS treatment, respectively (Figure 3A). Macrophage and lung epithelial cells secrete lysozyme that hydrolyze glycosidic bond of peptidoglycan which can damage the mycobacterial cell wall. In presence of lysozyme (500 ug/ml), *M. s*_Rv1515c showed nine-fold increase in survival as compared to the *M. s*_Vc (Figure 3B).

*M. tb*, being an intracellular pathogen, encounters acidic pH environment in the lysosomes of the macrophage. The effect of Rv1515c in conferring resistance to acidic stress was studied. In presence of mild acid (pH 5), *M. s*_Rv1515c showed significantly (*p* < 0.01) higher survival as compared to the *M. s*_Vc at 24 h. A nearly 1.5 fold increased survival was observed in *M. s*_Rv1515c after 48 h as compared to the *M. s*_Vc (Figure 3C). These results suggest that Rv1515c enables the mycobacteria to adapt to the intra-macrophage acidic environment**.**

The role of Rv1515c in conferring drug resistance was also studied *in vitro* by culturing the bacteria in presence of anti-TB drugs (Figure 3, Supplementary Figure S3). *M. s*_Rv1515c exhibited greater resistance to the anti-TB drugs as compared to *M. s*_Vc. The minimum inhibitory concentration (MIC) was calculated to be 4 and 8 μg/ml for ethambutol and streptomycin, respectively against *M. s*_Rv1515c (Figure 3D). This indicates that Rv1515c enhances resistance to frontline anti-TB drugs and may confer protection to the *M. tb*.

Since Rv1515c modulates cell morphology, a decrease in susceptibility to antibiotics could also be due to alteration in cell wall permeability. We studied the accumulation of Nile Red (hydrophobic) and EtBr (hydrophilic) in *M. s*_Vc and *M. s*_Rv1515c cells. Our results indicated that the uptake of Nile Red and EtBr was slow in *M. s*_Rv1515c as compared to the *M. s*_Vc. Over a period of 1.5 h, lower levels of Nile Red and EtBr accumulated in *M. s*_Rv1515c as compared to the *M. s*_Vc (Figure 3E). Rv1515c plays a key role in decreasing the permeability of the cell wall which decreases penetration or uptake of stress factors including drugs that may aid in survival of the *M. tb* within the host.

### *M. s*_Rv1515c Inhibits Oxidative Stress and Arrests Late Apoptosis to Promote Bacterial Survival in the Macrophages

**FIGURE 4:**
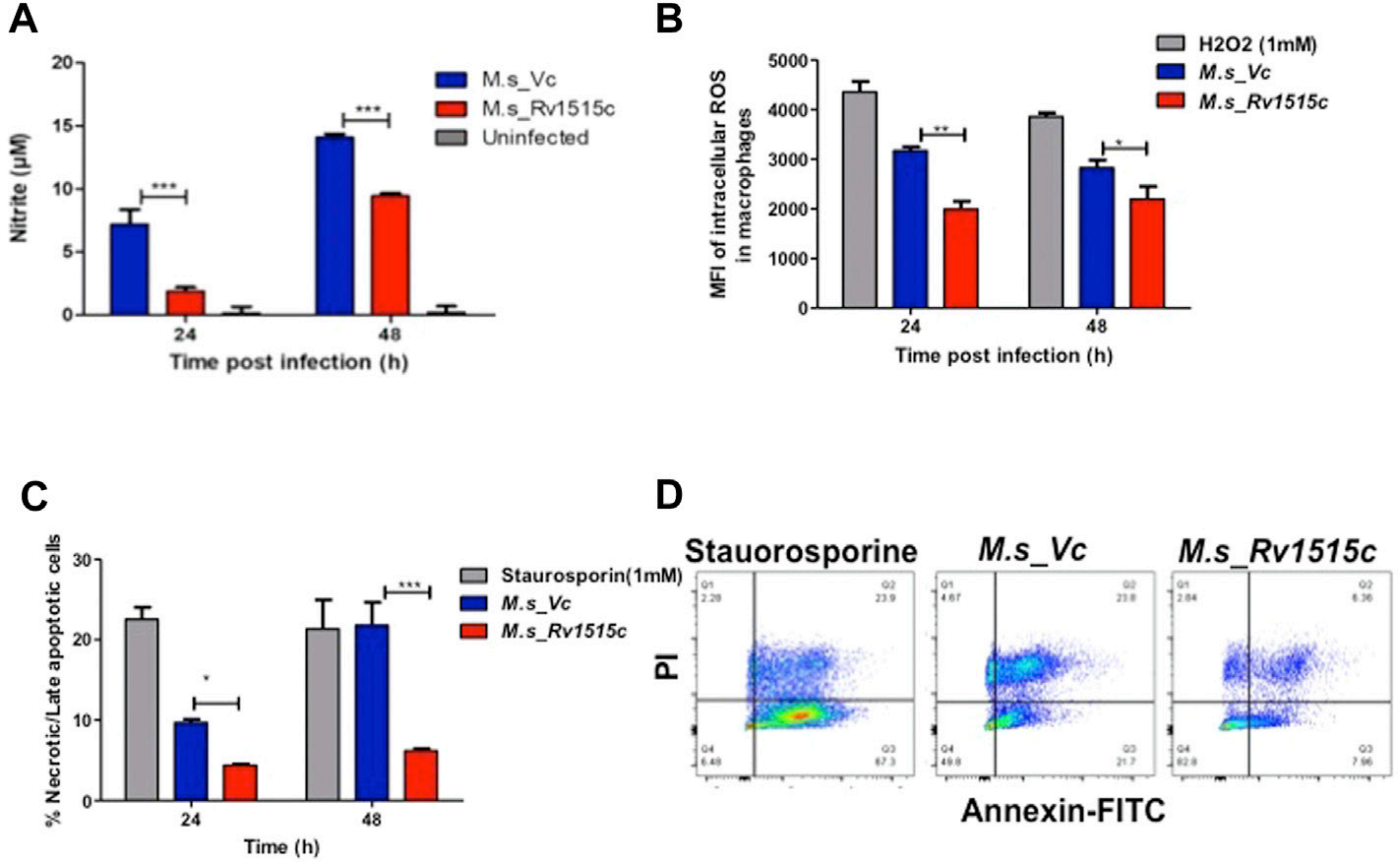
Rv1515c decreases the production of ROS and NO, and arrests apoptosis in macrophages. RAW 264.7 cells (0.5 × 10^6^ cells) were incubated with equal number of *M. s*_Vc and *M. s*_Rv1515c cells at a MOI of 1:10 for 24 and 48 h. **(A)** Griess reagent was added to the culture supernatant and level of NO was measured spectrophotometrically at 545 nm. Uninfected cells were used as control. **(B)** ROS level was analysed by flow cytometry post staining with CellROX Green dye. Mean fluorescent intensity (MFI) of intracellular ROS production in infected macrophages. H_2_O_2_ was used as a positive control. **(C)** Annexin-PI assay was used to assess percent apoptotic cells by flow cytometry, as described in methods. Staurosporine treated cells were used as positive control. **(D)** Representative scatter plot of apoptosis assay at 48 h. Data represent mean ± SD of at least three independent experiments. Statistical analysis was performed using two-way ANOVA followed by Bonferroni test. *p < 0.05, **p < 0.01, ***p < 0.001.

In order to assess the role of Rv1515c in conferring protection to the bacteria residing within the macrophages, RAW264.7 cells were infected with *M. s*_Rv1515c and *M. s*_Vc at MOI of 1:10. The supernatant was collected from infected macrophages at 24 and 48 h post-infection and reactive nitrogen species (RNS) were quantified by determining nitric oxide (NO) level using Griess reagent kit. ROS level was also quantified flow cytometrically by CellROX Green Reagent assay. It was observed that *M. s*_Rv1515c infected macrophage produced significantly (*p* < 0.001) lower levels of NO (Figure 4A) and ROS (Figure 4B) as compared to *M. s*_Vc infected macrophage. Knock-in of *Rv1515c* gene in *M. smegmatis* aids the pathogen to downregulate host ROS and RNS, thereby enabling the bacteria to survive in macrophages.

*M. tb* downregulates apoptosis in order to survive within the host cells. To assess the role of Rv1515c in apoptosis, RAW264.7 macrophages were infected with *M. s*_Rv1515c and *M. s*_Vc at MOI of 1:10. The extracellular bacteria that failed to invade the macrophage after 4 h of co-infection were washed off using DMEM containing gentamycin. Percent apoptotic cells were estimated after 24 and 48 h using FITC Annexin V apoptosis detection kit. We observed a significant decrease in apoptosis in macrophages infected with *M. s*_Rv1515c as compared to the *M. s*_Vc (Figures 4C,D). We also assessed the expression of (voltage-dependent anion-selective channel protein-1 (VDAC1) that triggers apoptosis by directing the release of apoptosis-causing proteins through an oligomeric channel (Shoshan-Barmatz et al., 2017; Shoshan-Barmatz et al., 2020). Rv1515c suppressed the expression level of VDAC1 in RAW 264.7 macrophages infected with *M. s*_Rv1515c compared to *M. s*_Vc (Supplementary Figure S4A). This result suggests that Rv1515c lowers the VDAC1 expression level to suppress the pro-apoptotic response to inhibit apoptosis in macrophages to favour mycobacterial survival within host macrophages. These result suggests that Rv1515c arrests apoptosis, thereby aiding the mycobacteria to survive within the macrophage.

### Rv1515c Aids in Virulence and Survival of Mycobacteria Within the Macrophages by Enabling the Pathogen to Escape Phago-Lysosomal Fusion

**FIGURE 5:**
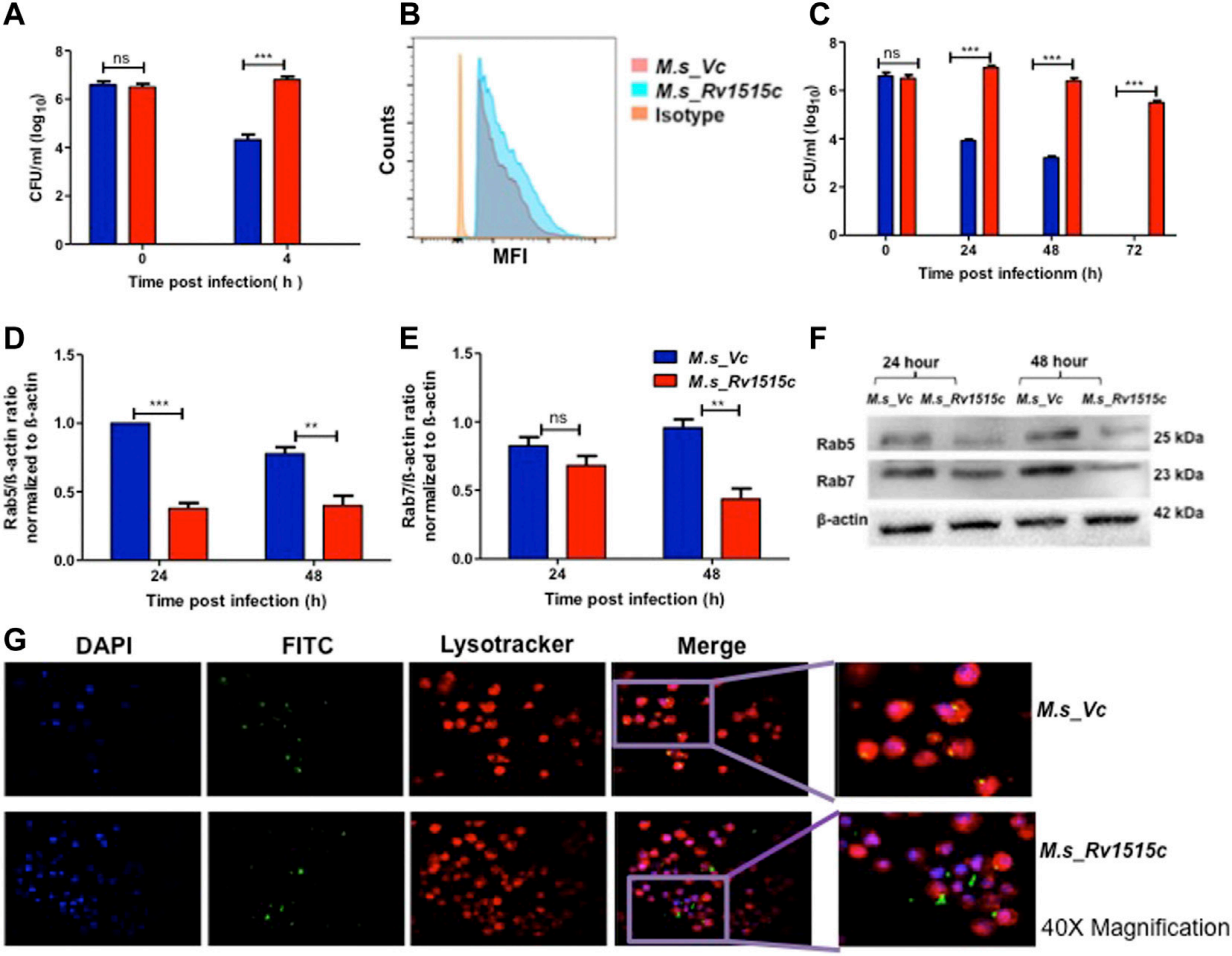
Recombinant *M. smegmatis* expressing *M. tb* Rv1515c exhibit increased uptake and survive by escaping the phagolysosomal fusion. RAW264.7 cells were incubated with equal number of *M. s*_Vc and *M. s*_Rv1515c at MOI of 1:10 for 4 h. Extracellular bacteria were washed off and RAW 264.7 cells were re-seeded/incubated in culture media. **(A)** Bacteria that were phagocytized in 4 h of co-infection were enumerated by CFU assay, as described in methods. **(B)** Representative flow cytometry data show enhanced uptake of *M. s*_Rv1515c. **(C)** RAW 264.7 cells were lysed at 0, 24, 48, and 72 h post re-seeding to extract the intracellular bacteria that survived. Bacteria was plated on LB agar and CFU assay was done. **(D)** Expression of early endosomal marker Rab5 is downregulated by *M. s*_Rv1515c. **(E)** Late endosomal marker Rab7 is downregulated by *M. s*_Rv1515c. **(F)** Representative western blot image of Rab5, Rab7, and β-actin (control) expression in infected RAW264.7 macrophages at 24 and 48 h post-reseeding/incubation. **(G)** Representative fluorescence microscopy (at 40X magnification) images depicting phagolysosomal fusion of *M. s*_Vc and phagolysosomal escape of *M. s*_Rv1515c bacteria in infected macrophages at 72 h post-infection. Inset: the enlarged image of respective treatments. Data are represented as means of at least three independent experiments, and statistical analysis was performed using two-way ANOVA followed by Bonferroni test. ***p < 0.001.

The role of Rv1515c in virulence and pathogenicity were assessed *in vitro* by assessing the invasiveness and survival of *M. s*_Rv1515c within the macrophage, as described in methods. After 4 h of co-infection with macrophages, extracellular *M. s*_Rv1515c and *M. s*_Vc were washed off. CFU count of bacteria that were internalized in macrophages exhibited nearly two-fold higher uptake of *M. s*_Rv1515c as compared to the *M. s*_Vc cells (Figure 5A). Flow cytometric data also showed significantly (*p* < 0.001) higher phagocytosis of FITC labelled *M. s*_Rv1515c as compared to the *M. s*_Vc (Figure 5B). This result indicated the potential role of *Rv1515c* gene in *M. tb* virulence. Knock-in of *M. tb Rv1515c* gene in non-pathogenic *M. smegmatis* imparted enhanced ability to the recombinant strain in undergoing phagocytosis in macrophages, indicating the role of Rv1515c in establishing infection in the host cells.

Macrophages containing intracellular bacteria were re-seeded in culture plate and examined after 72 h for bacterial survival by CFU count and microscopically. CFU count showed a significantly (*p* < 0.001) higher survival of *M. s_Rv1515c* (up to two fold) within the macrophage as compared to *M. s*_Vc (Figure 5C). *M. s*_Rv1515c could survive within the macrophage up to 72 h whereas *M. s_Vc* failed to survive. Cell lysates from macrophages, infected with *M. s*_Rv1515c and *M. s*_Vc, were collected at 24 and 48 h post infection. Western blot analysis of early and late phagosomal markers Rab 5 and Rab7 proteins, respectively were done. It was found that Rab 5 expression (Figures 5D,F) was significantly downregulated at 24 and 48 h; and Rab 7 expression (Figures 5E,F) was decreased at 48 h. Rv1515c arrests late stage of phagosomal maturation. Microscopic examination also showed that *M. s*_Rv1515c survived up to 72 h inside RAW 264.7 macrophages, whereas *M. s*_Vc could survive only up to 48 h post-infection. Recombinant *M. smegmatis* expressing Rv1515c escaped phagosomal compartments (Figure 5G, Supplementary Figure S5) of macrophages, thereby favouring the mycobacteria to survive. Cathelicidin antimicrobial peptide (CAMP) favours the intracellular survival of pathogen in macrophages. Hence, we assessed the expression of CAMP encoded cathelicidin related antimicrobial peptide (CRAMP) in RAW 264.7 cells infected with *M. s*_Rv1515c and *M. s*_Vc. The level of CRAMP was downregulated in RAW 264.7 cells infected with *M. s*_Rv1515c as compared to the vector control (Supplementary Figure S4B). These results showed that Rv1515c may aid *M. tb* survival within the host cell and cause pathogenesis.

### Rv1515c Suppresses Pro-Inflammatory Response and Molecules Involved in Antigen Presentation for Evading the Host Immune Response

**FIGURE 6:**
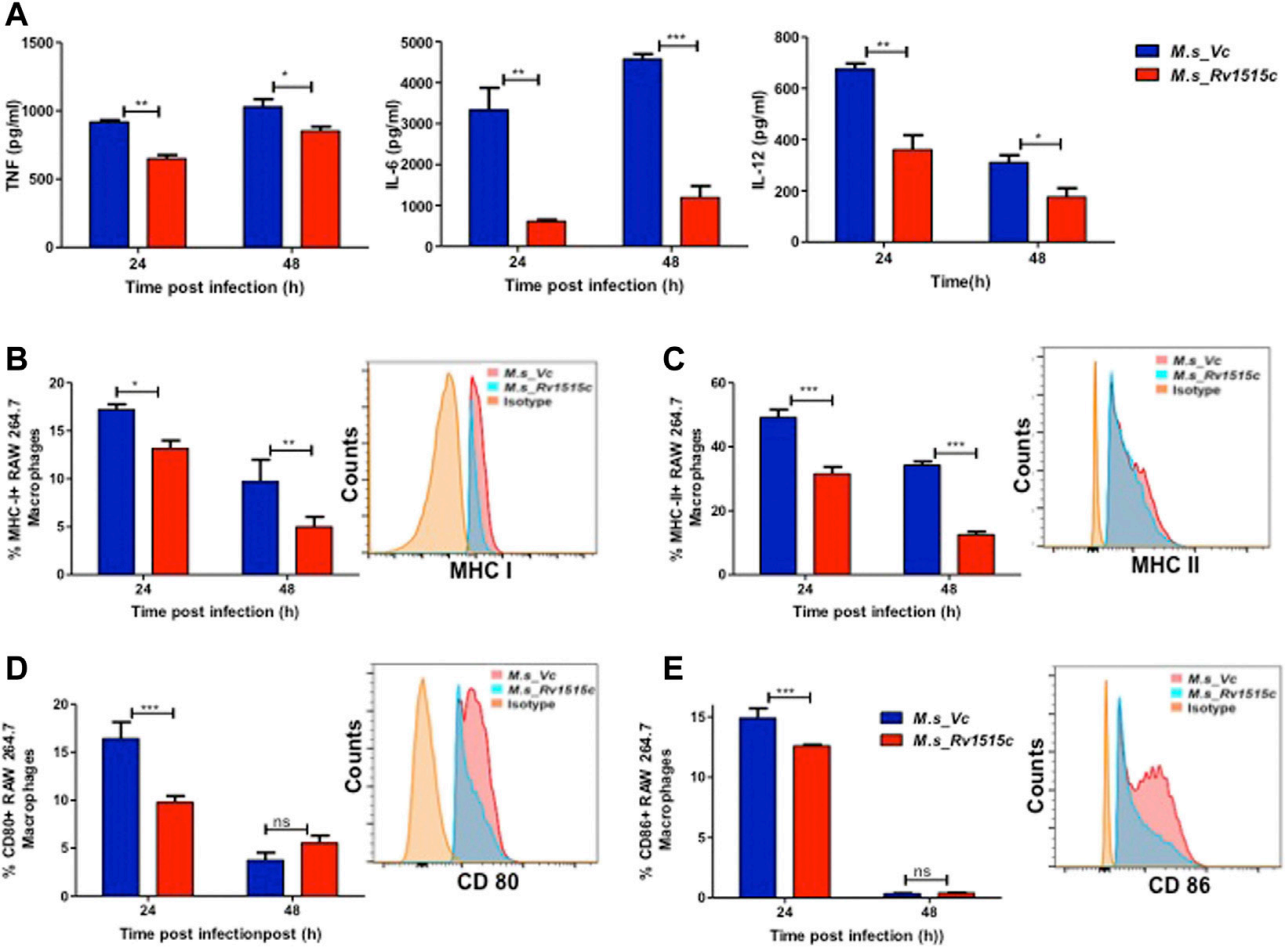
Recombinant *M. smegmatis* expressing *M. tb* Rv1515c suppress pro-inflammatory cytokine and molecules involved in antigen presentation. RAW 264.7 cells were incubated with equal number of *M. s*_Vc and *M. s*_Rv1515c cells at a MOI of 1:10 for 24, 48 h. **(A)** The culture supernatant was collected and ELISA was done to assess the levels of cytokines: TNF-α, IL-6, and IL-12. Percent cells expressing **(B)** MHC I, **(C)** MHC II, **(D)** CD80, **(E)** CD86 in the culture were analysed flow cytometrically. Inset shows the representative histogram of flow cytometry at 24 h. Data are represented as mean ± SD from three independent experiments, and statistical analysis was performed using two-way ANOVA followed by Bonferroni test. *p < 0.05, **p < 0.01, ***p < 0.001.

Host immune response plays a crucial role in preventing the pathogen to establish infection. The role of Rv1515c in modulating the immune response was assessed by infecting RAW264.7 macrophage cells with *M. s*_Rv1515c and *M. s*_Vc. The cytokine levels in macrophage culture supernatants were estimated using sandwich ELISA. At 24 h, *M. s*_Rv1515c infection significantly (*p* < 0.01) suppressed the production of IL-6 (8-fold decrease) and IL-12 (2-fold decrease). The level of TNF-α was also significantly (*p* < 0.05) reduced upon infection with *M. s*_Rv1515c as compared to *M. s*_Vc. At 48 h, infection with *M. s*_Rv1515c led to significant (*p* < 0.001) reduction in IL-6 (5-fold decrease), reduction (*p* < 0.05) in IL-12 and TNF-α level, as compared to *M. s*_Vc (Figure 6A). The expression of TNF receptors and tumour necrosis factor alpha-converting enzyme (TACE) were examined in RAW 264.7 cells treated *M. s*_Rv1515c and *M. s*_Vc. Real-time PCR results (Supplementary Figure S6) showed that Rv1515c significantly downregulated mRNA expression of TNFR1, and TNFR2 in macrophages, indicating low turnover of TNF-α levels. TACE level was also significantly (*p* < 0.001) decreased at 48 h, which correlates the production of TNF-α in infected macrophages with *M. s*_Rv1515c compared to *M. s*_Vc. These results pointed that Rv1515c suppresses pro-inflammatory response which may aid the *M. tb* to subvert host immune response.

Intracellular pathogens such as *M. tb* are processed in macrophages through the MHC pathway which presents truncated antigens of the pathogen for recognition by T cells, leading to activation of T cells. Our *in vitro* results showed that *M. s*_Rv1515c infection caused suppression of antigen presentation by macrophages, as is evident by a significant reduction in the expression of MHC I (*p* < 0.01) (Figure 6B) and MHC II (*p* < 0.001) (Figure 6C). *M. s*_Rv1515c also significantly inhibits the expression co-stimulation markers CD80 (Figure 6D) and CD86 (Figure 6E). Rv1515c may aid *M. tb* to dampen host defense mechanisms for antigen recognition and may favour the pathogen to survive within the macrophages.

### Rv1515c Induces CD4^+^ Treg Cells That Suppress Generation of T Cells in the Lymph Nodes Which Cause Decreased Clearance of the Mycobacteria *In Vivo*

**FIGURE 7:**
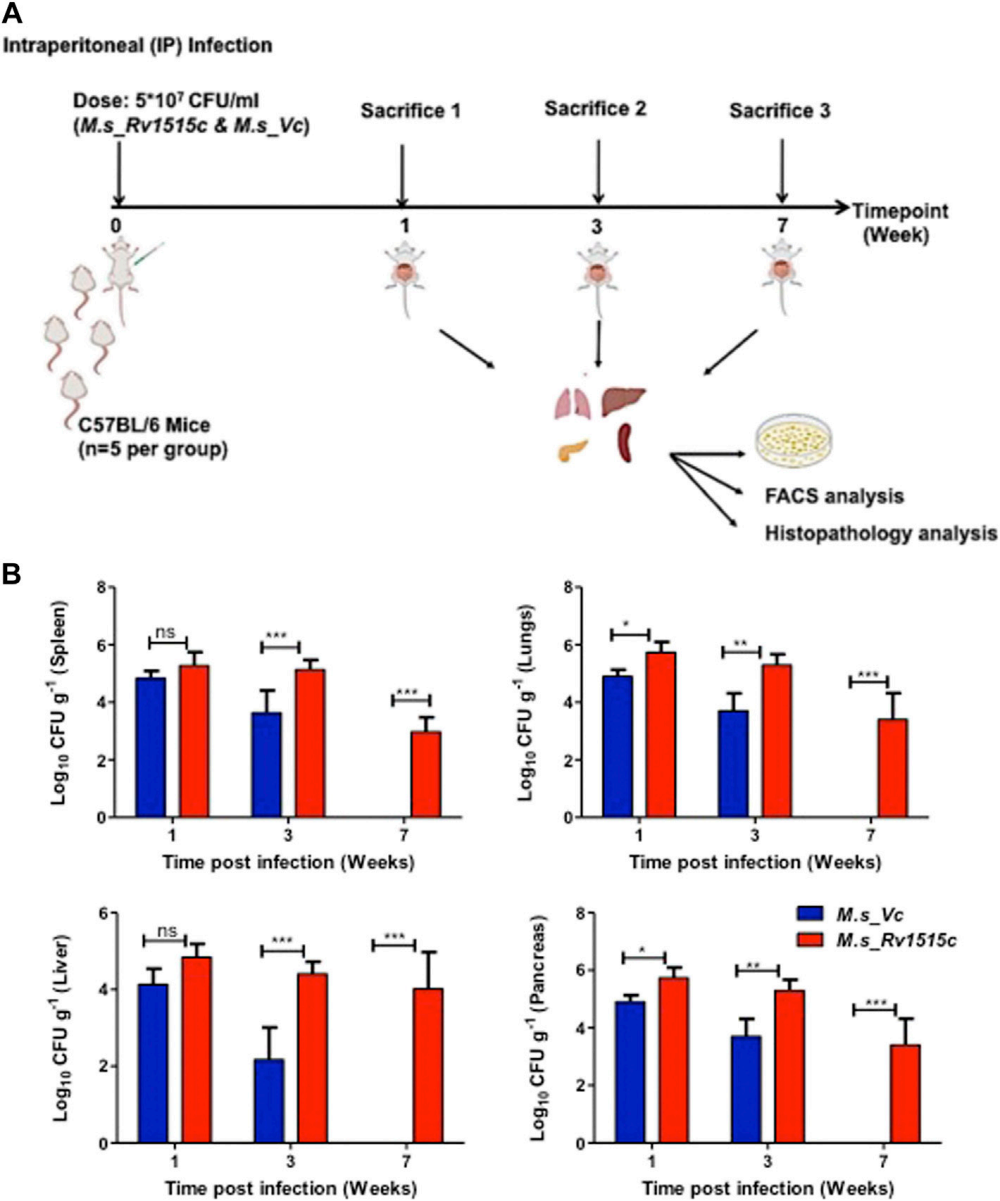
Recombinant *M. smegmatis* expressing *M. tb* Rv1515c persists *in vivo*. **(A)** Schematic representation of experiments to assess the effect of Rv1515c *in vivo*. C57BL/6 female mice (*n* = 6) were infected intraperitoneally with *M. s*_Rv1515c and *M. s*_Vc. The bacterial loads in the liver, lungs, spleen, and pancreas were determined after 1, 3, and 7 week post-infection. Organs were homogenized and bacterial load was assessed by CFU assay, as described in methods. Single cell suspension of the lymph node or intraperitoneal macrophages were stained with different markers for lymphocyte sub-populations and analysed by flow cytometry. **(B)** CFU counts of the *M. s*_Rv1515c and *M. s*_Vc that persisted in the spleen, lungs, liver, and pancreas were determined. Data are represented as mean ± SD and analysed using two-way ANOVA followed by Bonferroni test. *p < 0.05, **p < 0.01, ***p < 0.001, ns- nonsignificant.

C57BL/6 mice were infected intraperitoneally with *M. s*_Rv1515c and *M. s*_Vc, and the bacterial load in various organs were determined at three different time points: 1, 3, and 7 week post-infection (Figure 7A). After first week post-infection, the liver, kidney, spleen, and pancreas showed presence of bacteria. Bacterial burdens were significantly higher in the liver, lungs, spleen, and pancreas of mice infected with *M. s*_Rv1515c as compared to the *M. s*_Vc at one and 3 weeks post-infection. Furthermore, *M. s*_Rv1515c survived up to 7 weeks in the infected mice while *M. s*_Vc was completely cleared (Figure 7B).

**FIGURE 8:**
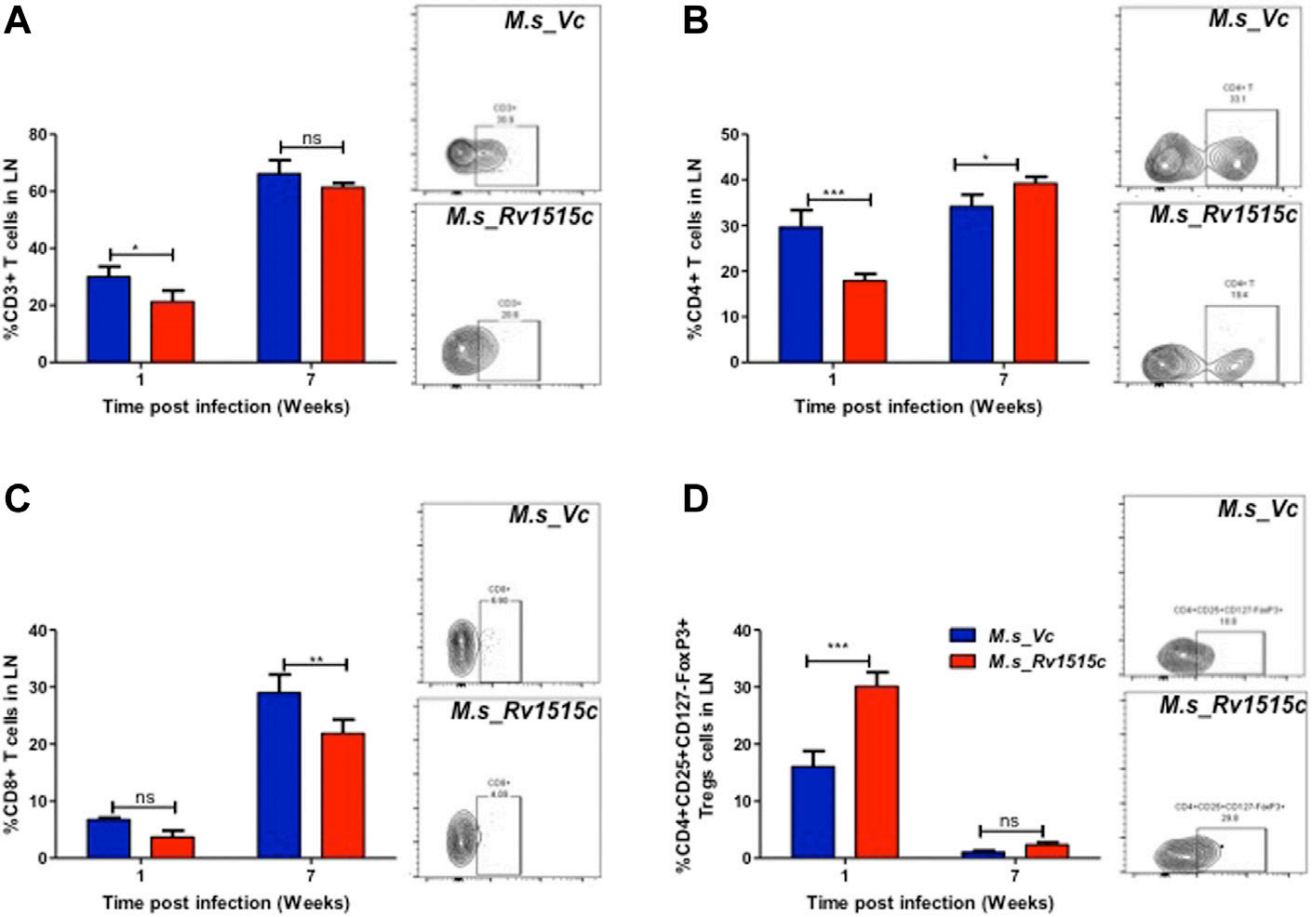
Rv1515c suppresses T effector response *via* upregulation of immunosuppressive Tregs. C57BL/6 female mice (*n* = 6) were infected intraperitoneally with *M. s*_Rv1515c and *M. s*_Vc. After 1 and 7 weeks post infection, lymph nodes were extracted and single cell suspensions were stained with antibodies to detect the percent of T cell sub-populations. **(A)** CD3^+^T cells, **(B)** CD4^+^T cells, **(C)** CD8^+^T cells, **(D)** Treg cells expressing CD4^+^CD25^+^CD127^−^FoxP3^+^. Inset: representative dot-plot of flow cytometry data for 24 h time point. Data are represented as mean ± SD and statistical analysis was performed using two-way ANOVA followed by Bonferroni test. *p < 0.05, **p < 0.01, ***p < 0.001, ns- nonsignificant.

T cell sub-populations play crucial role in preventing infection and clearance of the pathogens. In order to assess the possible role of Rv1515c in modulating the T cell response, T cell profiling in the lymph node of infected mice was carried out after 1 and 7 week post-infection with *M. s*_Rv1515c or *M. s*_Vc. After 1 week of infection, *M. s*_Rv1515c infection caused significant (*p* < 0.05) decrease in CD3^+^ T cells (Figure 8A) and 2-fold decrease in CD4^+^ T cells (Figure 8B). There was no significant decrease in CD8^+^ T cells, as compared to infection with *M. s*_Vc (Figure 8C). After 7 weeks of infection, *M. s*_Rv1515c caused significant decrease in CD8^+^ T cells (*p* < 0.01), as compared to infection with *M. s*_Vc. The CD4^+^ T cells were significantly (*p* < 0.05) increased in mice infected with *M. s*_Rv1515c.

The generation of T cells is regulated by the Treg cells, which express CD4^+^CD25^+^CD127^−^FoxP3^+^ markers (Yu et al., 2012). In order to understand the dynamics of T cell subpopulation regulated through Treg cells, we examined the role of Rv1515c in modulating Treg cells. After 1 week of infection, the percent of Treg cells from the lymph node was 2-fold higher in mice infected with *M. s*_Rv1515c, as compared to *M. s*_Vc (Figure 8D). After 7 weeks of infection, Treg cells reduced in numbers and there was no significant difference in mice infected either with *M. s*_Rv1515c or *M. s*_Vc. Enhancement of Treg cells suppress T cell generation, whereas decrease in Treg cells cause proliferation of T cells. Rv1515c upregulates Treg cells during initial phase of infection which suppress T cell generation, whereas during the later phase of infection Treg cells are downregulated which allows the CD4^+^ T cells to proliferate. During *M. tb* infection, Rv1515c may suppress T effector response *via* upregulation of immunosuppressive Tregs which may favour survival of the bacteria.

**FIGURE 9:**
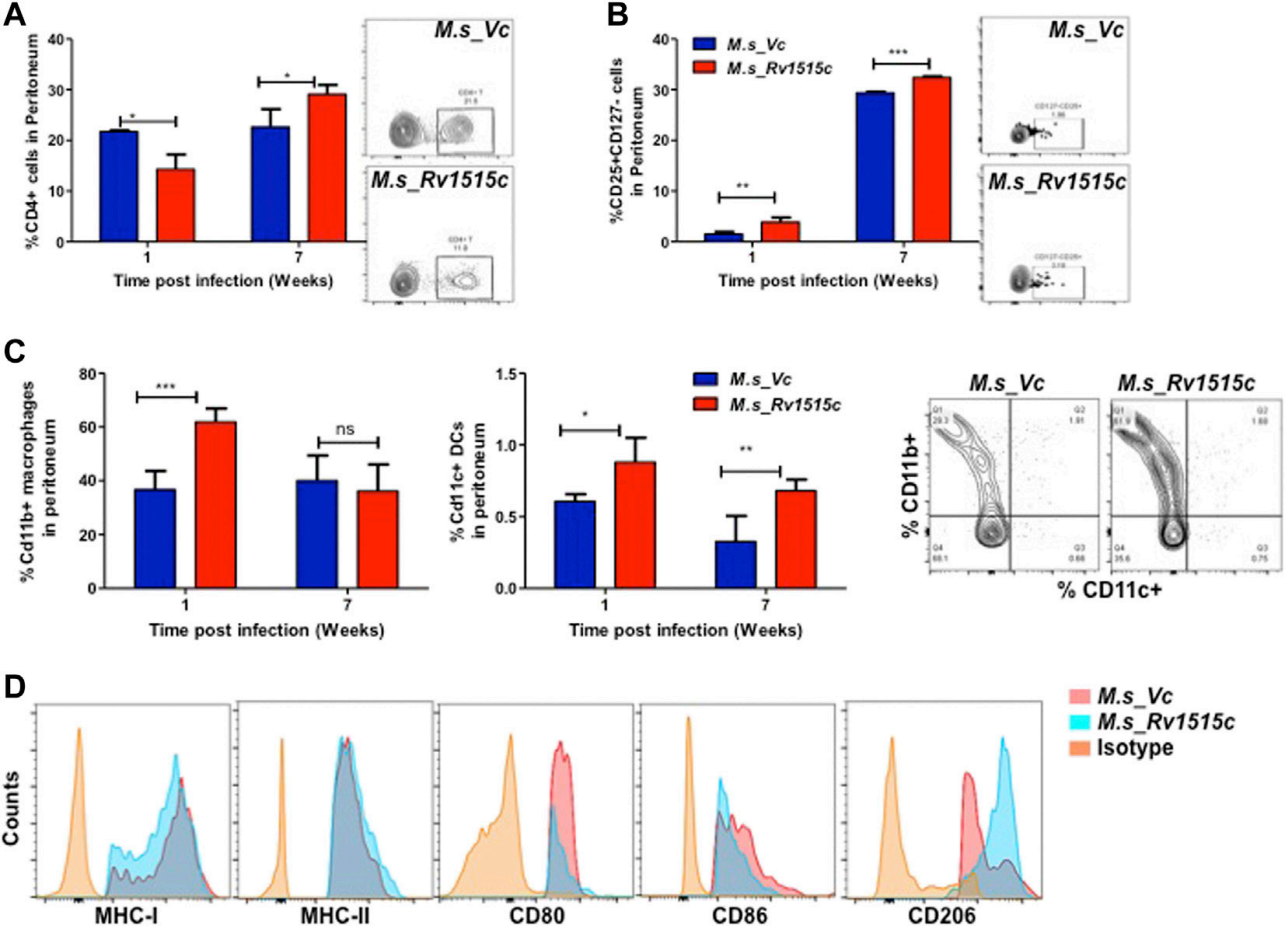
Phenotypic profiling of peritoneal APCs after infection with recombinant *M. smegmatis* expressing *M. tb* Rv1515c. C57BL/6 female mice (*n* = 6) were infected intraperitoneally with *M. s*_Rv1515c and *M. s*_Vc. After 1 and 7 weeks post infection, the peritoneal macrophages were extracted and single cell suspensions were stained with antibodies to detect the percent of T cell sub-populations. **(A)** CD4^+^ T cells; **(B)** Percent Treg cell expressing of CD25^+^CD127^−^ on gated CD4^+^ T cells were examined; **(C)** CD11b^+^ expressing macrophages and CD11c^+^ expressing dendritic cells. Inset: representative dot plot of the CD11b^+^ CD11c^+^ cells at 1 week time point. **(D)** Expression of antigen presentation molecules (MHCI, MHCII), co-stimulatory molecules (CD80, CD86) and M2-macrophage polarization marker CD206. Data are represented as mean ± SD and statistical analysis was performed using two-way ANOVA followed by Bonferroni test. *p < 0.05, **p < 0.01, ***p < 0.001, ns- nonsignificant.

Peritoneal lymphocytes are among the first responding cells that act on infections that occur in the peritoneal cavity. Since *M. s*_Rv1515c and *M. s*_Vc were intraperitoneally administered to the mice, we examined the effect of Rv1515c on the dynamics of peritoneal lymphocytes. There was a significant (*p* < 0.05) decrease in CD4^+^ T cell 1 week post-infection, which later on increased significantly (*p* < 0.05) after 7 weeks in mice infected with *M. s*_Rv1515c (Figure 9A). Treg cells increased significantly (*p* < 0.05) in mice infected with *M. s*_Rv1515c as compared to *M. s*_Vc up to 7 weeks post-infection (Figure 9B) in the peritoneum of infected mice with recombinant *M. smegmatis* (Figure 9B). A nearly 2-fold increase in peritoneal CD11b^+^ macrophages was observed in mice after 1 week of infection with *M. s*_Rv1515c (Figure 9C). Our results also showed that CD11c^+^ dendritic cells numbers increased in the peritoneal cavity of the mice after 1 week of infection with *M. s*_Rv1515c. After 7 weeks, although the percent of CD11c^+^ dendritic cells out of the total lymphocyte populations decreased in mice, infection with *M. s*_Rv1515c showed 2-fold higher number of dendritic cells as compared to the mice infected with *M. s*_Vc. These results suggest that Rv1515c may modulate the T cell dynamics. MHC I expressing antigen presenting cells was reduced in the peritoneal cavity of mice infected with *M. s*_Rv1515c for 1 week as compared to *M. s*_Vc. In contrast, the same group of mice showed increased MHC II expressing antigen presenting cells in the peritoneal cavity. The costimulatory molecules, CD80 and CD86, were suppressed due to infection with *M. s*_Rv1515c, pointing to the role of Rv1515c in dampening the costimulatory signals required for T cell activation (Figure 9D). CD206^+^ expressing M2-macrophages were significantly increased in the peritoneal cavity of the mice infected with *M. s*_Rv1515c (Figure 9D), which suggested that the anti-inflammatory role of M2-macrophages may cause bacterial persistence especially in the organs localized in the peritoneal cavity. Rv1515c thus modulates the homeostasis of peritoneal lymphocytes that may favour *M. tb* to survive within the host.

### Histopathological Changes in Mice Infected With Recombinant *M. smegmatis* Expressing *M. tb* 1515c

**FIGURE 10:**
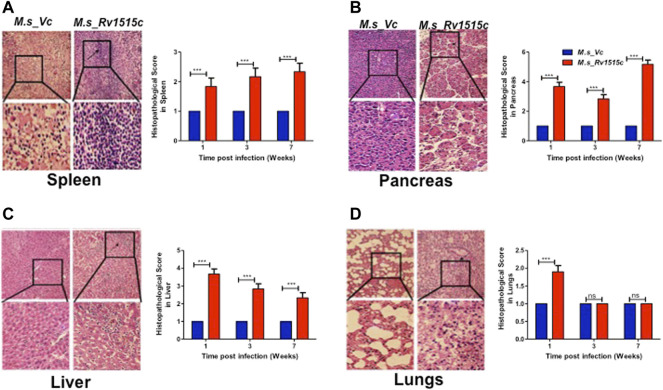
Histopathological analysis of major organs in mice post-infection with *M. s_Vc* and *M. s_Rv1515c*. C57BL/6 female mice (*n* = 6) were infected intraperitoneally with *M. s*_Rv1515c and *M. s*_Vc. Organs were extracted, fixed and stained with H and E stain, as described in methods. Representative histopathological images (of 1 week post infection) for the various organs at 20X magnification was taken. **(A)** Spleen- extramedullary haematopoiesis (EMH) and distorted spleen architecture (indicated by arrow) post-infection with *M. s*_Rv1515c. **(B)** Pancreas- acinar cells destruction and lymphocytic infiltration (indicated by arrows) in pancreas. **(C)** Liver- lymphocytic infiltration and inflammation in the liver of mice infected with *M. s*_Rv1515c. **(D)** Lungs- mild haemorrhage in alveolar and parenchymal region of lung at 1 week (indicated by arrows). The histopathological parameters were blindly scored on samples obtained at 1, 3, and 7 weeks, and scored as 1 = Normal histology, 2 = Mild changes, 3 = Moderate changes, 4 = Severe changes, 5 = Highly severe change. Bar graph shows the histopathology score as mean ± SD and analysed using two-way ANOVA followed by Bonferroni test. ***p < 0.001, ns- nonsignificant.

Our results showed that Rv1515c suppresses lymphocyte populations which may delay *M. tb* clearance from the host. Results in Figure 10 show the histopathological examination of the liver, spleen, pancreas, and lungs that were carried out in mice infected with *M. s*_Rv1515c or *M. s*_Vc for 7 weeks. We observed splenomegaly in mice infected with *M. s*_Rv1515c (Supplementary Figure S7). Spleen, obtained from mice infected with *M. s*_Rv1515c, exhibited mild follicular destruction, increased lymphocytic infiltration and extramedullary haematopoiesis, which increased over time. In the pancreas of mice infected with *M. s_*Rv1515*c*, mild acinar destruction, moderate inflammation and lymphocytic infiltration were observed at 1 and 3 weeks post-infection. A 5-fold increase in acinar cells destruction was observed in mice infected with *M. s*_Rv1515c as compared to the *M. s*_Vc. Liver cells in mice infected with *M. s*_Rv1515c showed higher lymphocytic infiltration, inflammation in the periportal and parenchymal regions, as compared to mice infected with *M. s*_Vc. Over the course of time, infiltration of the neutrophils in the liver reduced and only mild pathology was observed in mice infected with *M. s*_Rv1515c. In mice treated with *M. s*_Rv1515c, there was decrease in alveolar space and mild haemorrhage was observed in the alveolar and parenchyma region after 1 week of infection. These results show that Rv1515c may play a key role in enabling *M. tb* to establish infection in host tissue and cause pathology.

## Discussion

*M. tb* possesses a wide repertoire of virulence factors that enable it to infect the host and counteract the immune response. The interplay between various virulence factors allow the pathogen to adapt to challenge from the host immune response. DNA methylation is important for epigenetic imprinting in prokaryotes (Shell et al., 2013). Presence of methyl cytosine in only the virulent *M. tb* H_37_Rv strains but not in non-virulent H_37_Ra strains points to the crucial role of DNA methylation in *M. tb* virulence (Srivastava et al., 1981). S-adenosylmethionine (AdoMet)-dependent methylation is an important biochemical mechanism that involves the transfer of a methyl group from AdoMet to S-adenosylhomocysteine and a methylated molecular target. Several reports suggest that MTases are involved in regulating some of the key cellular functions. MamA, MamB, and HsdM are three known DNA MTases coded by *M. tb*, which target different sequence pattern for N6-adenine methylation (Shell et al., 2013; Zhu et al., 2016). Mycobacterial DNA methyltransferase HSdM regulates transcription and decreases susceptibility to isoniazid (Hu et al., 2021). Rv1988 is a secretory methyltransferase that enters the nucleus of the host and methylates histone H3 at arginine residues, regulating the expression of genes that neutralise reactive oxygen species (Yaseen et al., 2015). Rv2966c is a secretory methyltransferase that methylates cytosine and hijacks the epigenetic machinery of the host DNA. MamA regulates the expression of several genes involved in survival of *M. tb* during hypoxia (Shell et al., 2013). *M. tb tlyA* gene product Rv1694 has two distinct functions, haemolytic action and ribosomal RNA methylation. Haemolytic activity of Rv1694 has a role in the phagosomal compartment and likely aids in macrophage entry (Rahman et al., 2010). Rv1523 is involved in methylation of the mycobacterial cell envelope lipids and interacts with proteins involved in the fatty acid biosynthetic pathways. Since Rv1523 is involved in biosynthesis of lipids involved in cell wall, its activity is crucial to achieve resistance to antibiotics in *M. tb*. Nearly 71% of the *M. tb* MTases exhibit antigenicity and thus can also have a role in immunomodulation (Grover et al., 2016). Rv0470c, a SAM dependent MTase, is involved in cyclopropanation of mycolate and is implicated in virulence and persistence. In the course of the reductive genomic evolution of *M. tb* from non-pathogenic mycobacteria (Rahman et al., 2014), the acquisition of new gene Rv1515c through horizontal transfer may have implications in the emergence of the pathogenic lifestyle of *M. tb* (Alexander et al., 2009; Veyrier et al., 2009). The present study documents a functional role of the hypothetical protein Rv1515c in virulence, host immune-modulation and pathogenesis.

Rv1515c exhibits S-adenosyl methionine binding site and conserved MTase domain (Yang et al., 2021). Our study showed the physical interaction of the Rv1515c with DNA; and the MTase activity of Rv1515c was confirmed (Figure 1) by the ability of the Rv1515c to transfer a methyl group from Adomet to cytosine, resulting in methylation of cytosine residue on DNA. DNA MTases play an important role in epigenetic modifications that regulates several key cellular functions including transcription factors, cell signalling, virulence factors etc. *M. tb* being a slow grower, we elucidated the functional role of the hypothetical protein Rv1515c by cloning the *M. tb Rv1515c* gene in the non-pathogenic fast growing strain of *M. smegmatis* and confirmed the expression of Rv1515c in recombinant *M. smegmatis*. Rv1515c being exclusive to *M. tb*, any alterations in the physiological properties in the recombinant strains of *M. smegmatis* expressing Rv1515c (*M. s*_Rv1515c) could be attributed to the hypothetical protein Rv1515c.

Knock-in of *M. tb Rv1515c* gene in *M. smegmatis* resulted in changes in morphology and surface topology of the recombinant *M. s*_Rv1515c. The *M. s*_Rv1515c showed altered colony morphology and rough surface architecture which could be due to modifications in the cell surface. Pertinent to this, Rv1515c is capable of localising to the mycobacterial cell wall and cell membrane. SEM and AFM analysis revealed that *M. s*_Rv1515c cells had reduced bacterial cell length and wider diameter as compared to control *M. smegmatis*. TEM analysis (Figure 2) showed that *M. s*_Rv1515c exhibited granularity and the overall thickness of the cell membrane was significantly increased. Our study showed that *M. s*_Rv1515c forms aggregates in the aqueous solution and the hydrophobicity of the recombinant strain increases, indicating change in the composition of the cell membrane. A study by Yang et al. (2021) showed that Rv1515c modulates expression of several lipids including 7- heptenyl-lauric acid (C12:1ω7c), 3-methyl-hexadecanoic acid (3MeC16:0), nonoic acid (C9:0), and undecanoic acid (C11:0). Previous studies reported that altered cell surface hydrophobicity and cell wall morphology correlated with lipid modifications in the *mycobacterium* cell envelope (Kansal et al., 1998; Chen et al., 2006; Singh et al., 2016). *M. tb* MTases Rv1523, mmaA4, etc. play an important role in modulating the mycobacterial cell wall mycolic acids (Dao et al., 2008; Ali et al., 2021). Colony morphology is affected by the cell wall composition. *M. tb* colony is characterized by rough and irregular surfaces that aggregate to form clumps. While non-pathogenic *M. smegmatis* forms smooth colonies, the recombinant *M. s*_Rv1515c formed clumps suggesting the functional role Rv1515c in modulating the cell wall phenotype and virulence. Previous studies have highlighted that mycobacterial clumping is an important factor for conferring virulence which increases the ability of the bacteria to damage the macrophages (Brambilla et al., 2016; Mahamed et al., 2017).

Several *M. tb* MTases such as Rv1523, Rv0560 etc. play an important role in conferring resistance to the environmental stress, protection from drugs and host immune responses (Ali et al., 2021). In order to elucidate the role of hypothetical protein Rv1515c, *in vitro* cultures of recombinant *M. s*_Rv1515c were treated with 0.1% SDS, mild acid (pH 5), lysozyme, and anti-tubercular drugs. Our studies showed that Rv1515c expression conferred protection to the recombinant *M. s*_Rv1515c strain in stress conditions such as that faced by the *M.tb* within the macrophage. We observed that *M. s*_Rv1515c strain showed decreased sensitivity (Figure 3D) to anti-TB drugs (Ethambutol and streptomycin). TEM analysis of *M. s*_Rv1515c showed increased thickness of the cell wall which may prevent the penetration of the drugs or stress causing molecules. The modulation in cell permeability due to activity of Rv1515c was spectrophotometrically confirmed by assays for uptake of Nile red or EtBr (Figure 3E) in the recombinant *M. s*_Rv1515c strains. The roles of other MTase such as Rv0560c in conferring drug resistance have been reported earlier (Gamngoen et al., 2018). Decreased sensitivity to anti-TB drugs correlated with reduced cell wall permeability. Rv1515c can thus confer drug tolerance to *M. tb*, thereby allowing it to adapt to the hostile environment in the host cell.

Macrophages deploy defense mechanisms such as production of ROS, under the influence of NOX2 and reactive nitrogen species (RNS) to generate oxidative stress, thereby providing a inconducive environment for the pathogen (Miller et al., 2010). *M. tb* has evolved several factors that suppress formation of ROS and reactive nitrogen species thereby allowing it to establish an intracellular niche for survival (Koster et al., 2017; Arora et al., 2020; Manjunath et al., 2021). Our study showed that in RAW264.7 macrophages cells infected with *M. s*_Rv1515c, there is a significant decrease in the production of NO (Figure 4A) and ROS (Figure 4B). Rv1515c downregulates macrophage defense mediators which favours survival of mycobacteria within the macrophage.

Macrophage can undergo apoptosis to prevent the spread of infectious pathogen. As a counter-mechanism *M. tb* residing within the macrophage can induce apoptosis and necrosis in order to disseminate (Behar et al., 2011; Aguilo et al., 2013; Augenstreich et al., 2017; Dallenga et al., 2017; Lerner et al., 2017; Shariq et al., 2021; Sharma et al., 2021) from the host cells. Yang et al. (2021) incubated recombinant strain of *M. smegmatis* expressing Rv1515c with THP-1 cells for 24 h to show increased apoptosis in cells. We tried to revisit the effect of Rv1515c in inducing apoptosis in macrophage. In our assay, we co-cultured the recombinant strain of *M. smegmatis* (*M. s*_Rv1515c) with RAW264.7 macrophages for 4 h. Subsequently, we washed off the extracellular *M. s*_Rv1515c that failed to invade the RAW264.7 macrophages with media containing gentamycin. This ensured that the apoptosis recorded in our assay is due to the intracellular bacteria only that could invade the macrophage within the initial 4 h. If the extracellular bacteria are allowed to remain in the co-culture media they may cause a possible artifact in the apoptosis assay. Contrary to the study by Yang et al. (2021), we observed that intracellular *M. s*_Rv1515c suppressed apoptosis of macrophage cells. Suppression of macrophage apoptosis is favourable for the intracellular bacteria as it can persist within the niche of cell for longer duration (Danelishvili et al., 2010). We believe that Rv1515c suppresses apoptosis in the macrophage so that the bacteria can survive within the cell. This is in line with our observations that showed that Rv1515c expressing *M. s*_Rv1515c can survive for as long as 72 h (Figure 5C) while *M. smegmatis* failed to survive within the macrophage. Several mycobacterial components arrest apoptosis by inhibiting ROS signalling pathway and contribute to virulence and pathogenesis of *M. tb* (Miller et al., 2010; Paik et al., 2019). Rv1988, a histone methyltransferase present only in *M. tb*, altered the expression of the genes necessary for ROS production to hijack the host immune system which favours bacterial survival (Yaseen et al., 2015; Khosla et al., 2016; Shen et al., 2020). On similar lines, the knock-in of Rv1515c in *M. smegmatis* resulted in inhibition of oxidative stress *via* diminishing ROS and NO production in the macrophages. Cathelicidin (LL-37) is involved in controlling the intracellular survival of mycobacteria in macrophages (Sonawane et al., 2011). Vitamin D also protects against TB through “nonclassical” processes such as the induction of antimicrobial peptides LL-37 (Martineau et al., 2007). Previous study reported that rapid upregulation of cathelicidin antimicrobial peptide CAMP is associated with early resistance to non virulent mycobacterial infecrtion in mice (Adam et al., 2018). In our study, CAMP encoding cathelicidin related antimicrobial peptide (CRAMP) level was downregulated in RAW 264.7 macrophages infected with *M. s*_Rv1515c than vector control. The low expression of CAMP favours the intracellular survival of recombinant *M. smegmatis* in macrophages.

Macrophages phagocytose the pathogens and engulf them within the lysosomal compartments, thereby killing the pathogens. However, *M. tb* hijacks this mechanism of macrophage to gain entry into the macrophage through phagosome compartments and survive intracellularly by subverting lysosome-phagosome fusion. We observed that there is enhanced uptake of recombinant *M. s*_Rv1515c as compared to control *M. s*_Vc cells in the macrophage. The increased invasiveness of the *M. s*_Rv1515c indicates that Rv1515c plays a role in virulence. We also observed that while control *M. s*_Vc cells were cleared within the lysosomal compartment of the macrophages, *M. s*_Rv1515c survived within the macrophages for as long as 72 h (Figure 5C). This indicated that Rv1515c can impart persistence to the *M. tb* to survive within the macrophage and may have a role in *M. tb* latent infections. While the macrophage possess mechanisms to channelize the endocytosed pathogens to the lysosomal compartments, *M. tb* has evolved mechanisms which inhibits phagosomal acidification and maturation, or escape the phagolysosomal fusion (Ehrt and Schnappinger, 2009; Carranza and Chavez-Galan, 2019). *M. tb* possesses several virulence factors such as Rv0297 which inhibits phagosomal acidification by downregulating the expression of the Rab7 marker (Sharma et al., 2020). We studied the effect of Rv1515c on phagolysosomal fusion and observed that Rv1515c downregulates early and late endosome formation as evident by decreased expression of Rab5 (Figures 5D,F) and Rab 7 (Figures 5E,F), respectively. Our results demonstrates that *M. s*_Rv1515c expressing Rv1515c escapes phagolysosomal fusion (Figure 5G), indicating its mechanistic role in conferring survival advantage to the *M. tb* within the macrophage.

The ability of the pathogen to cause infection levers on the interplay between the virulence factors of pathogens and the host immune response. Host immune cells release cytokines such as TNF-α, IL-6, and IL-12 that cause inflammatory signals which recruit more effector cells such as macrophages, neutrophils etc. to site of infection, thereby clearing the pathogen. On the other hand, the pathogen has to subvert host immune response in order to establish infection. Our study showed that Rv1515c downregulated the secretion of pro-inflammatory cytokines TNF-α, IL-6, and IL-12, which may dampen the host immune response, thereby allowing the pathogen to survive. Increased survival of *M. s*_Rv1515c in presence of macrophage correlates with the downregulation of pro-inflammatory cytokines TNF-α, IL-6, and IL-12 due to the activity of Rv1515c. The TNF receptors are associated with increased inflammatory response and reduced bacterial clearance, which leads to widespread mycobacterial infection and host cell death (Chavez-Galan et al., 2016; Ruiz et al., 2021). Mannose-capped *M. tb* lipoarabinomannan stimulates the synthesis of soluble tumour necrosis factor receptors by activating the tumour necrosis factor alpha-converting enzyme (TACE) to promote TNF neutralization (Richmond et al., 2012). Real-time PCR was performed to check TNFR1, TNFR2, and TACE expression levels in RAW264.7 macrophage post-infection with *M. s*_Vc and *M. s*_Rv1515c. Decreased levels of TNFR1, TNFR2, and TACE correlated with decreased levels of TNF response. By suppressing the pro-inflammatory cytokine, Rv1515c can inhibit macrophage activation which may favour *M. tb* pathogenesis.

Antigen presenting cells (APCs) including macrophages play a key role in activation of effector T cells. The endocytosed pathogen is degraded in a complex antigen processing mechanism into smaller peptides which are presented through the MHC I or MHC II to the CD8^+^ or CD4^+^ T cells, respectively. The recognition of antigens by T cells is important to establish a cascade of T cell proliferation which allows efficient clearance of pathogen. Studies have shown that *M. tb* PPE38 protein inhibits the expression of MHC I and diminished the number of CD8^+^ T cells to escape host immune defence (Meng et al., 2017). The role of hypothetical protein Rv1515c in modulating the antigen processing mechanism was studied *in vitro*. Our results showed that *M. s*_Rv1515c expressing Rv1515c suppresses the expression of MHC I and MHC II on macrophage. Decreased expression of MHC I and MHC II could offset the process of recognition of pathogen by T cells which may allow recurrent infections. The cytotoxic activity of the CD8^+^ T cells is required for clearance of intracellular pathogens and activation of T helper cells is required to activate the cascade of immune response. By suppressing the MHC I, MHC II, CD80, and CD86 *M. tb* is able to override the host defense mechanism generated by T cells (Cooper 2009; Sakai et al., 2014; Hmama et al., 2015).

Microarray analysis of gene expression in the lungs during the chronic phase of *M. tb* infection in the mice showed that *Rv1515c* was upregulated (Gautam et al., 2015). *In vivo* experiments showed that mice infected with *M. s*_Rv1515c exhibited higher bacterial load in the major organs studied, such as the spleen, liver, pancreas, and the lungs. Increased persistence of *M. s*_Rv1515c in the infected mice for longer time duration validated our *in vitro* data. Mice infected with *M. s*_Rv1515c showed acute histopathological symptoms during the early and chronic phase of infections. These studies suggest that Rv1515c is an important factor that may confer pathogenicity to *M. tb*.

Previous studies have shown that *M. tb* can impair the Th1 mediated response through increased activity of the regulatory T cell (Treg) (Geffner et al., 2009; Fan et al., 2016; Davids et al., 2018). Several studies have reported a higher Treg level in TB patients than in the healthy control (Pang et al., 2013; Luo et al., 2017). The immunosuppressive role of Treg can be detrimental to the host and provide an advantage to the pathogen (Cardona and Cardona, 2019). We observed elevated level of Treg in the lymph nodes and peritoneum of mice infected with *M. s*_Rv1515c as compared to the *M. s*_Vc, thus highlighting the role of Rv1515c in TB pathogenesis. Rv1515c induced higher level of Treg that suppressed the effector T cell response by downregulating the CD4^+^ and CD8^+^ T cells. Our results on mice challenged with *M. s*_Rv1515c show reduction in CD25^+^FoxP3^+^/CD127^low^ Treg cells. It is known that Treg cells delay the arrival of effector T cells at the site of infection to impede protective immunity in the host (Shafiani et al., 2010). Adoptive transfer of CD25^+^T cells to *M. tb* infected mice favours bacterial survival (Okeke and Uzonna, 2021). Thus, Tregs mediated reduction in IFN-γ production, as a mechanism to suppress anti-*M. tb* immunity, can be restored following transient Tregs depletion in TB patients (Hougardy et al., 2007; Whittaker et al., 2017). Research shows that frequency of Tregs cells are higher in MDR-TB and XDR-TB patients than individuals with drug-sensitive or latent TB (Li et al., 2015; Fan et al., 2016; Davids et al., 2018). Thus, modulating Treg levels by targeting *M. tb* virulence factors, including Rv1515c, may augment clearance of *M. tb*.

**FIGURE 11:**
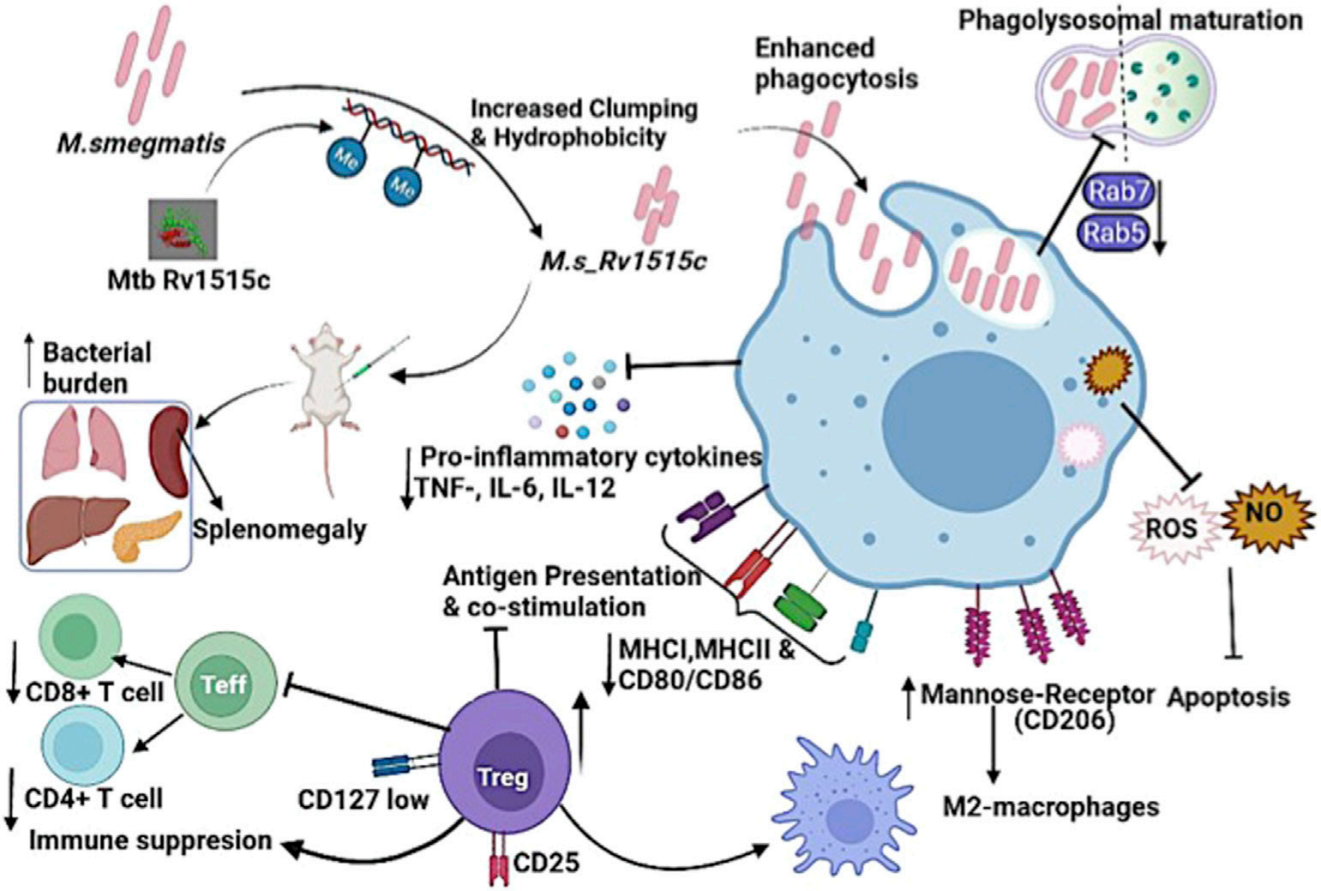
Model displaying the distinct role of *M. tb* methyltransferase Rv1515c in host-immune modulation: Knock-in of *M. tb Rv1515c* gene in *M. smegmatis* (*M. s*_Rv1515c) altered cell dimension, morphology and cell surface hydrophobicity. The recombinant *M. s*_Rv1515c expressing Rv1515c protein is more virulent and is phagocytosed by the macrophage. *M. s*_Rv1515c exhibits higher survival within the macrophage due to its ability to escape phagolysosomal fusion. The early and late phagolysosomal markers Rab5 and Rab7, respectively is downregulated. Rv1515c dampens the production of reactive oxygen species (ROS) and nitric oxide (NO) within the macrophage and suppresses apoptosis, which favors mycobacterial persistence in the macrophages. Rv1515c aids the bacteria to resist immune response. Rv1515c suppresses the molecules involved in antigen presentation (MHCI, MHCII) and co-stimulation (CD80 and CD86). Rv1515c suppresses effector T cell response *via* upregulation of immunosuppressive Tregs cells which may favour bacterial survival.

The CD11b^+^ peritoneal macrophages populations increased in mice after 1 week of infection with *M. s*_Rv1515c and CD11^+^c dendritic cells were not tolerized but increased after 7 week post-infection in mice administered with *M. s*_Rv1515c as compared to *M. s*_Vc. However, despite the flux of APCs in peritoneum (site of infection) the priming of T cells was significantly reduced in Rv1515c infected animals which suggests possible defects in presentation and processing of antigens in lymph nodes (LNs), similar to the previous reports (Shafiani et al., 2010). Our results thus establish Rv1515c as a virulence factor of *M. tb* which help prolong the intra-host bacterial residency by impairing efficient priming and mobilization of effector T cells during the early phase of infection. *M. tb* has evolved strategies to escape killing by the M1 activated macrophage and switch macrophage to the M2 state to promote chronic infection in the host (Lastrucci et al., 2015; Queval et al., 2016). Activated M2a macrophages express mannose receptor (CD206) and differentiate under the influence of Th2 induced cytokines. M2 macrophages downregulate the pro-inflammatory immune response (Fraternale et al., 2015; Thiriot et al., 2020). Interestingly, M2 macrophage marker CD206 was highly expressed in mice infected with recombinant *M. s*_Rv1515c, signifying that Rv1515c has anti-inflammatory role to suppress host immune response that can inhibit pathogen clearance. These results correlate with exacerbated tissue pathology observed in extrapulmonary organs of mice infected with *M. s*_Rv1515c. In summary, our study highlighted the role of hypothetical protein Rv1515c in conferring virulence and pathogenesis to *M. tb*. Figure 11 summarises the functional role of the Rv1515c in host pathogen interaction and immune modulation. The possibility to exploit Rv1515c as a drug target for therapeutic interventions for TB is an open question and may be explored.

## Data Availability

The original contributions presented in the study are included in the article/Supplementary Material, further inquiries can be directed to the corresponding authors.

## Supplementary Material

Supplementary Figure S1 I-Tasers predicted model of *M. tb* Rv1515c protein. pBlast analysis and red ribbon structure displaying methyltransferase domain.

Supplementary Figure S2
**(A)** Growth kinetics *M. s*_Vc and *M. s*_Rv1515c cells. **(B)** Bacterial cells (*M. s*_Vc and *M. s*_Rv1515c) were kept in static condition for one hour. *M. s*_Rv1515c forms clumps in aqueous solution and settles at the bottom.

Supplementary Figure S3 Effect of Isoniazid in survival of bacteria (*M. s*_Vc and *M. s*_Rv1515c). Growth of *M. s*_Vc and *M. s*_Rv1515c in presence of serial dilutions of anti TB drug Isoniazid was estimated.

Supplementary Figure S4 Rv1515c suppresses VDAC1 and antimicrobial peptide cathelicidin (CRAMP). RAW264.7 macrophages were infected with *M. s*_Rv1515c or *M. s*_Vc at a MOI 1:10 . After 24 and 48 h post infection, real-time PCR analysis was performed to detect fold changes inexpression of VDAC1 and CRAMP. The levels of A) VDAC1; B) CRAMP mRNA changes over time. The relative fold change of transcripts was calculated using the 2^ (−ΔΔCT) method with reference to the housekeeping gene β-actin. 2^ (−ΔΔCT) values and mean ± SD are used to represent data from three independent experiments and statistical analysis was performed using two-way ANOVA followed by Bonferroni posttest. ***p* < 0.001.

Supplementary Figure S5 The phagolysosomal fusion of *M.s*_Vc and phagolysosomal escape of *M. s*_Rv1515c bacteria in infected macrophages at 72 h post-infection are shown as bar graph.

Supplementary Figure S6 Rv1515c downregulates expression of TNFR1, TNFR2, and TACE. RAW264.7 macrophages were infected with *M. s*_Rv1515c or *M. s*_Vc at a MOI 1:10. RAW 264.7 cells were examined using real-time PCR analysis to detect fold changes in TNFR1, TNFR2, and TACE. The levels of A) TNFR1, B) TNFR2, C) TACE mRNA changes in RAW264.7 cells were infected with *M. s*_Rv1515c or *M. s*_Vc. The relative fold change of transcripts was calculated using the 2^ (−ΔΔCT) method to calculate the relative fold change of transcripts level with reference to house keeping gene β-actin. Data are represented as by 2^ (−ΔΔCT) values with mean ± SD of three independent experiments and statistical analysis was performed using two-way ANOVA followed by Bonferroni posttest. ****p* < 0.001, ns, non significant.

Supplementary Figure S7 Splenomegaly was observed in mice infected with recombinant *M. s*_Rv1515c as compared to *M. s*_Vc.

Supplementary Table S1 List of primers used for RT-PCR reactions.
