# Supplementary figures and images for: Mycobacterium tuberculosis Methyltransferase Rv1515c Can Suppress Host Defense Mechanisms by Modulating Immune Functions Utilizing a Multipronged Mechanism
Source: Front Mol Biosci. 2022 Jun 24;9:906387. doi: 10.3389/fmolb.2022.906387 (PMC9263924; doi:10.3389/fmolb.2022.906387)

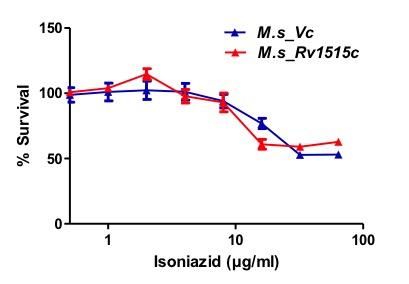

Supplement: Supplementary file 1 [file Image3.JPEG]

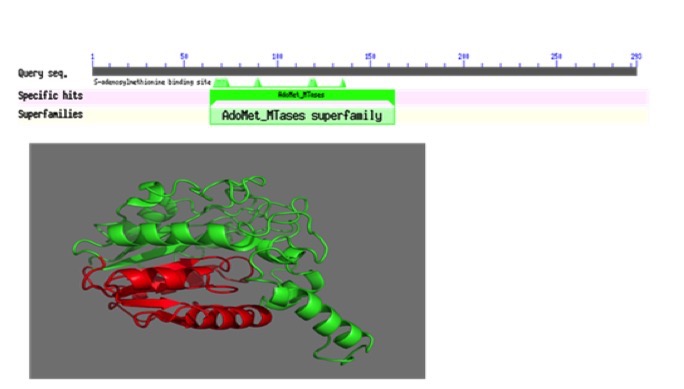

Supplement: Supplementary file 3 [file Image1.JPEG]

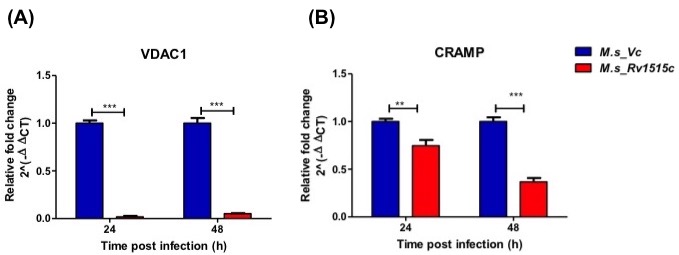

Supplement: Supplementary file 4 [file Image4.JPEG]

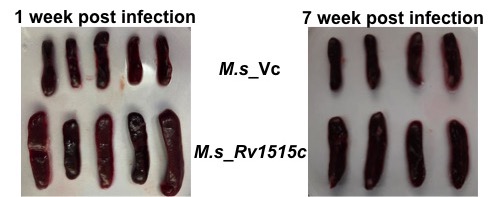

Supplement: Supplementary file 5 [file Image7.JPEG]

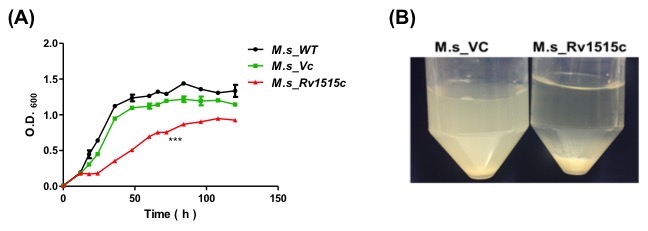

Supplement: Supplementary file 6 [file Image2.JPEG]

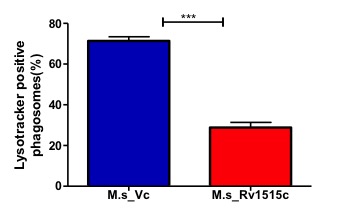

Supplement: Supplementary file 7 [file Image5.JPEG]

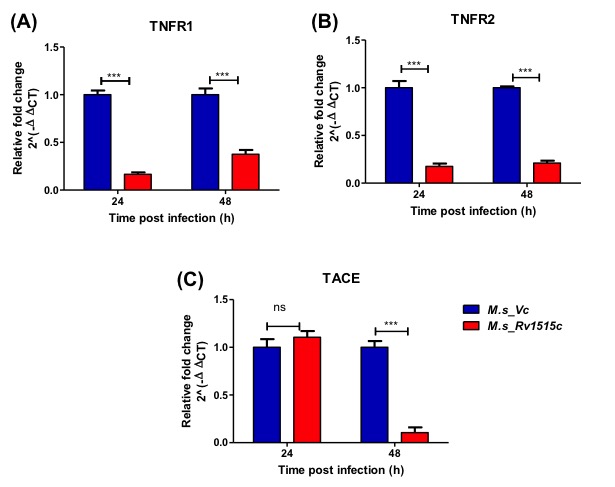

Supplement: Supplementary file 8 [file Image6.JPEG]
