# Supplementary material for: Mycobacterium tuberculosis Methyltransferase Rv1515c Can Suppress Host Defense Mechanisms by Modulating Immune Functions Utilizing a Multipronged Mechanism
Source: Front Mol Biosci. 2022 Jun 24;9:906387. doi: 10.3389/fmolb.2022.906387 (PMC9263924; doi:10.3389/fmolb.2022.906387)
Supplement: Supplementary file 2 [file Table1.pdf]

Supplementary Table 1: Lists of the primers used for real time-PCR

**Gene**	**Forward primer**	**Reverse Primer**
β-actin	CTGTCCCTGTATGCCTCTG	ATGTCACGCACGATTTCC
TNFR1	CAGTCTGCAGGGAGTGTGAA	CACGCACTGGAAGTGTGTCT
TNFR2	TACCAAGGGTGGCATCTCTC	TCCTGGGATTTCTCATCAGG
TACE	TGTGGTTATTTAAATGCAGATAGTGA	TCACTCGACGAACAAACTCTTC
CRAMP	AAGGAACAGGGGGTGGTG	CCGGGAAATTTTCTTGAACC
VDAC1	AATGACGGGACAGAGTTTGG	AATGACGGGACAGAGTTTGG
